# High-dimensional profiling reveals phenotypic heterogeneity and disease-specific alterations of granulocytes in COVID-19

**DOI:** 10.1073/pnas.2109123118

**Published:** 2021-09-21

**Authors:** Magda Lourda, Majda Dzidic, Laura Hertwig, Helena Bergsten, Laura M. Palma Medina, Indranil Sinha, Egle Kvedaraite, Puran Chen, Jagadeeswara R. Muvva, Jean-Baptiste Gorin, Martin Cornillet, Johanna Emgård, Kirsten Moll, Marina García, Kimia T. Maleki, Jonas Klingström, Jakob Michaëlsson, Malin Flodström-Tullberg, Susanna Brighenti, Marcus Buggert, Jenny Mjösberg, Karl-Johan Malmberg, Johan K. Sandberg, Jan-Inge Henter, Elin Folkesson, Sara Gredmark-Russ, Anders Sönnerborg, Lars I. Eriksson, Olav Rooyackers, Soo Aleman, Kristoffer Strålin, Hans-Gustaf Ljunggren, Niklas K. Björkström, Mattias Svensson, Andrea Ponzetta, Anna Norrby-Teglund, Benedict J. Chambers, Karolinska KI/K COVID-19 Study Group

**Affiliations:** ^a^Center for Infectious Medicine, Department of Medicine Huddinge, Karolinska Institutet, Karolinska University Hospital, 141 52, Stockholm, Sweden;; ^b^Childhood Cancer Research Unit, Department of Women’s and Children’s Health, Karolinska Institutet, 171 77, Stockholm, Sweden;; ^c^Theme of Children’s Health, Karolinska University Hospital, 171 76, Stockholm, Sweden;; ^d^Department of Infectious Diseases, Karolinska University Hospital, 171 77, Stockholm, Sweden;; ^e^Division of Infectious Diseases, Department of Medicine Solna, Karolinska Institutet, 171 77, Stockholm, Sweden;; ^f^Division of Infectious Diseases and Dermatology, Department of Medicine Huddinge, Karolinska Institutet, 141 52, Stockholm, Sweden;; ^g^Department of Physiology and Pharmacology, Section for Anesthesiology and Intensive Care, Karolinska Institutet, 171 77, Stockholm, Sweden;; ^h^Function Perioperative Medicine and Intensive Care, Karolinska University Hospital, 171 77, Stockholm, Sweden;; ^i^Department of Clinical Interventions and Technology, Division for Anesthesiology and Intensive Care, Karolinska Institutet, 171 77, Stockholm, Sweden

**Keywords:** COVID-19, neutrophil heterogeneity, eosinophil and basophil activation, viral immune responses, high-dimensional flow cytometry

## Abstract

Since the outset of the COVID-19 pandemic, increasing evidence suggests that the innate immune responses play an important role in the disease development. A dysregulated inflammatory state has been proposed as a key driver of clinical complications in COVID-19, with a potential detrimental role of granulocytes. However, a comprehensive phenotypic description of circulating granulocytes in severe acute respiratory syndrome coronavirus 2 (SARS-CoV-2)−infected patients is lacking. In this study, we used high-dimensional flow cytometry for granulocyte immunophenotyping in peripheral blood collected from COVID-19 patients during acute and convalescent phases. Severe COVID-19 was associated with increased levels of both mature and immature neutrophils, and decreased counts of eosinophils and basophils. Distinct immunotypes were evident in COVID-19 patients, with altered expression of several receptors involved in activation, adhesion, and migration of granulocytes (e.g., CD62L, CD11a/b, CD69, CD63, CXCR4). Paired sampling revealed recovery and phenotypic restoration of the granulocytic signature in the convalescent phase. The identified granulocyte immunotypes correlated with distinct sets of soluble inflammatory markers, supporting pathophysiologic relevance. Furthermore, clinical features, including multiorgan dysfunction and respiratory function, could be predicted using combined laboratory measurements and immunophenotyping. This study provides a comprehensive granulocyte characterization in COVID-19 and reveals specific immunotypes with potential predictive value for key clinical features associated with COVID-19.

## Significance

Accumulating evidence shows that granulocytes are key modulators of the immune response to SARS-CoV-2 infection, and their dysregulation could significantly impact COVID-19 severity and patient recovery after virus clearance. In the present study, we identify selected immune traits in neutrophil, eosinophil, and basophil subsets associated with severity of COVID-19 and with peripheral protein profiles. Moreover, computational modeling indicates that the combined use of phenotypic data and laboratory measurements can effectively predict key clinical outcomes in COVID-19 patients. Finally, patient-matched longitudinal analysis shows phenotypic normalization of granulocyte subsets 4 mo after hospitalization. Overall, in this work, we extend the current understanding of the distinct contribution of granulocyte subsets to COVID-19 pathogenesis.

Caused by the novel severe acute respiratory syndrome coronavirus 2 (SARS-CoV-2), the current COVID-19 pandemic poses an unprecedented threat to the global health systems, with over 3 million confirmed deaths reported worldwide in May 2021 (https://www.who.int/emergencies/diseases/novel-coronavirus-2019). About 80% of COVID-19 patients present with a mild or moderate disease course, while 15 to 20% develop severe complications (1, 2), including respiratory failure, coagulation abnormalities, and life-threatening acute respiratory distress syndrome (ARDS).

A dysregulated inflammatory state has been proposed as a key driver of clinical complications of COVID-19 (3) and has been associated with increased mortality (4). High levels of several proinflammatory mediators (e.g., interleukin-1β [IL-1β], IL-6, tumor necrosis factor α [TNFα], and CXCL8) are detected early after viral infection (5), and the resolution of this inflammatory response seems impaired in patients with severe disease progression (5, 6). Many of these proinflammatory cytokines are associated with granulocyte activation and recruitment (7), and it has been reported that an increased neutrophil-to-lymphocyte ratio (NLR) in peripheral blood can be used as a prognostic marker of higher disease severity in COVID-19 patients, similar to SARS-CoV-1 and Middle East respiratory syndrome (8–10).

The cellular components of the polymorphonuclear granulocyte family—neutrophils, eosinophils, and basophils—arise from a common myeloid progenitor in humans (11) and protect against invading microbes. The role of granulocytes in viral infection has mostly been confined to neutrophils (12, 13), although eosinophils and basophils have been implicated in the host response to viruses as well (14, 15). In COVID-19, an increased neutrophil abundance in circulation is mirrored by a substantial enrichment of granulocytes in the inflamed lung (16, 17). Several reports highlighted the presence of immature neutrophils in the blood of COVID-19 patients (18, 19), potentially indicating the occurrence of emergency granulopoiesis in the bone marrow. The presence of immature neutrophil populations has been linked to a potential immunosuppressive role of neutrophils during SARS-CoV-2 infection (19, 20). A confounding factor in many of the reported studies is represented by the broad use of peripheral blood mononuclear cells (e.g., refs. 6, 18, and 21), where only the low-density granulocytic fraction can be captured, which could lead to an inherent bias in the result interpretation (22, 23). Longitudinal studies performed during the course of SARS-CoV-2 infection have revealed that neutrophil numbers slowly decrease during infection, and the rate of such decrease is lower in more-severe patients (6). Interestingly, basophil and eosinophil counts were found to be inversely correlated with neutrophil levels (24).

In the present study, we set out to delineate the phenotypic alterations within neutrophil, eosinophil, and basophil populations during the early phase of SARS-CoV-2 infection in patients with acute COVID-19 and in the convalescent phase. We provide a detailed analysis at the single-cell resolution of granulocyte diversity in fresh whole blood of COVID-19 patients using 25-color flow cytometry, integrated with soluble factor detection by proteomics and detailed clinical information. Overall, this study delineates the biological contribution of granulocyte subsets to COVID-19 pathophysiology, with an emphasis on their phenotypical heterogeneity in viral infection. The data reveal specific immunotypes with potential predictive value for key clinical features associated with COVID-19 severity.

## Results

### Altered Composition of Granulocyte Populations Is Associated with Disease Severity in COVID-19.

**Fig. 1.**
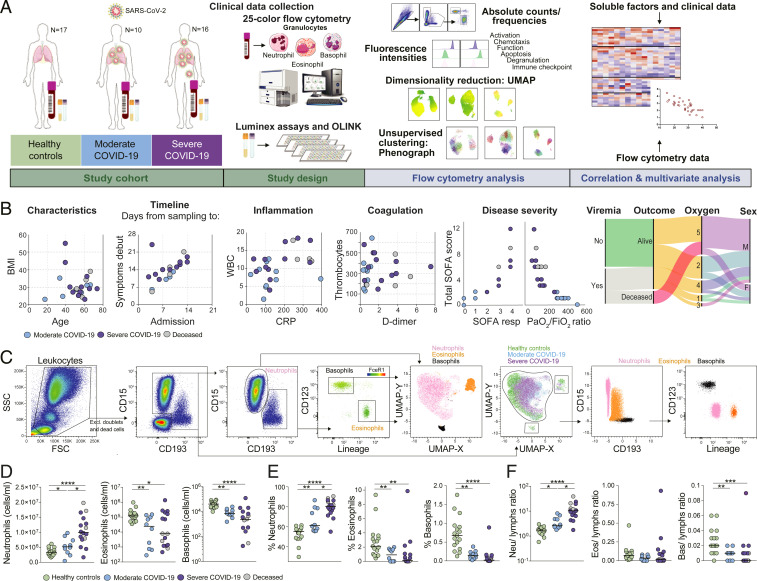
Disease severity-dependent neutrophilia and marked decrease of eosinophils and basophils in COVID-19. (*A*) Experimental and analytical workflow of the study performed on samples from moderate and severe COVID-19 patients and age-matched healthy controls. (*B*) Dot plots (*Left*) and alluvial diagram (*Right*) describing demographics and the clinical characteristics of the patients included in the study cohort. Individual patient values are reported in *SI Appendix*, Fig. S1*A*. Severity groups (moderate, blue; severe, purple; deceased, gray) are indicated. Oxygen scale: 1, no oxygen supply; 2, oxygen < 10 L/min; 3, low flow of oxygen (noninvasive) 10 L/min to 15 L/min; 4, high flow of oxygen (noninvasive); and 5, oxygen supply via ventilator (invasive) or extracorporeal membrane oxygenation. (*C*) Gating strategy for the identification of granulocyte subsets and their UMAP projection is shown on *Left*. On *Right*, analog results are obtained through unsupervised subset identification based on UMAP projection of total granulocytes. (*D* and *E*) Absolute cell counts (*D*) and frequencies (*E*), based on Trucount flow cytometry analysis, among total leukocytes for neutrophils, eosinophils, and basophils in healthy controls (*n* = 17), moderate COVID-19 patients (*n* = 10), and severe COVID-19 patients (*n* = 16). (*F*) Ratios of granulocyte absolute counts over lymphocyte absolute counts in healthy controls (*n* = 17), moderate COVID-19 patients (*n* = 10), and severe COVID-19 patients (*n* = 16). *D–F* use Kruskall−Wallis test and two-stage Benjamini, Krieger, and Yekutieli test. Bars represent median. *P < 0.05; **P < 0.01; ***P < 0.001; ****P < 0.0001. BMI, body mass index; WBC, white blood cells; CRP, C-reactive protein; SOFA, sequential organ failure assessment; SSC, side scatter; FSC, forward scatter; neu, neutrophils; eos, eosinophils; bas, basophils; lymphs, lymphocytes.

To provide an in-depth characterization of the granulocyte compartment in patients with moderate or severe COVID-19, we designed a strategy based on the integrated analysis of high-dimensional flow cytometry data from whole blood cells, extensive proteomic screening in serum and plasma, and detailed clinical and laboratory information from patients included in the study (Fig. 1*A*). Twenty-six hospitalized COVID-19 patients (10 moderate and 16 severe) and age-matched healthy controls were recruited at Karolinska University Hospital as part of the Karolinska KI/K COVID-19 Immune Atlas effort (25–28) (Fig. 1*B* and *SI Appendix*, Tables S1–S3 and Fig. S1 *A–D*). No differences in age, sex, body mass index (BMI), viremia, or time from symptom debut until hospital admission were present between moderate and severe patients (Fig. 1*B* and *SI Appendix*, Table S2 and Fig. S1 *A* and *C*). The severe group displayed higher concentration of the inflammatory markers D-dimer (severe: 2.76 ± 1.92 mg/L; moderate: 0.72 ± 0.33 mg/L) and C-reactive protein (CRP; severe: 196.18 ± 106.85 mg/L; moderate: 128.78 ± 106.23 mg/L), and coagulation and inflammatory markers, and included all patients (*n* = 4) who subsequently died in the hospital (Fig. 1*B* and *SI Appendix*, Fig. S1 *A* and *D*).

A 25-color flow cytometry panel was applied on freshly isolated whole blood cells from COVID-19 patients and healthy controls (Fig. 1*C*) to identify and characterize the main granulocyte subsets. To minimize batch effects, samples were processed and acquired during a short period of time (3 wk). A manual gating strategy for neutrophils, eosinophils, and basophils was used based on expression of canonical lineage-defining markers (Fig. 1*C*). Neutrophils were identified as CD15^+^CD193^−^CD66b^+^CD16^bright/dim^, eosinophils as CD15^bright/dim^CD193^bright/dim^CD16^−^FceR1^dim^CD123^dim/-^, and basophils as CD15^−^CD193^bright^FceR1^bright^CD123^bright^HLA-DR^dim/-^. Projection of gated cells into the uniform manifold approximation and projection (UMAP) space integrating all the analyzed markers confirmed the efficacy of the manual gating strategy and revealed that granulocytes from healthy controls clustered separately from patients with COVID-19, indicating potential alterations in the phenotype of the granulocyte compartment during SARS-CoV-2 infection (Fig. 1*C*).

The analysis of absolute leukocyte counts in COVID-19 patients highlighted relevant alterations in the granulocyte compartment in peripheral blood. In particular, neutrophils were significantly increased in patients with severe disease course, while eosinophils and basophils were decreased in COVID-19 patients when compared to noninfected healthy controls (Fig. 1*D*). A similar trend was observed in the relative frequencies of granulocyte subsets over total leukocytes. A higher proportion of neutrophils in patients with moderate and severe COVID-19 (66.65 ± 11.71% and 78.96 ± 8.83%, respectively) was associated with the reduction of the eosinophil and basophil fractions (up to threefold and 7.5-fold, respectively; Fig. 1*E*). The ratios of neutrophils over eosinophils or basophils increased in both patient groups, while no difference in the eosinophil to basophil ratios was observed between healthy controls and patients (*SI Appendix*, Fig. S1*E*). In line with previous reports (29), we found that severe COVID-19 was associated with higher NLRs and neutrophil-to-T cell ratios, as determined by absolute cell count (Fig. 1*F* and *SI Appendix*, Fig. S1*F*). We observed an opposite trend for basophils, which was associated with disease severity, while we did not detect any significant alteration in the eosinophil to lymphocyte or eosinophil to T cell ratios (Fig. 1*F* and *SI Appendix*, Fig. S1*F*).

This set of data highlights that the granulocyte expansion observed during acute SARS-CoV-2 infection is dominated by neutrophils and is coupled to the reduction of the physiological levels of circulating eosinophils and basophils.

### Severe COVID-19 Is Characterized by the Emergence of Immature Neutrophils in Peripheral Blood.

**Fig. 2.**
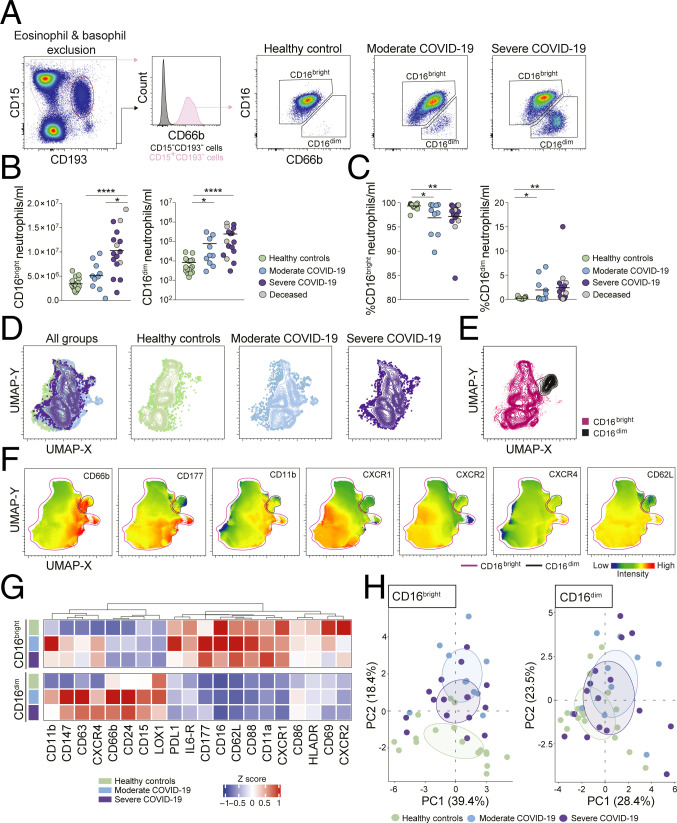
Emergence of immature neutrophils in severe COVID-19. (*A*) Representative plots showing neutrophil identification and CD16^bright^ and CD16^dim^ subset discrimination in different study groups. Absolute cell counts (*B*) and frequencies (*C*) among total neutrophils of CD16^bright^ and CD16^dim^ cells in healthy controls (*n* = 17), moderate COVID-19 patients (*n* = 10), and severe COVID-19 patients (*n* = 16). (*D*) UMAPs on concatenated files of total neutrophils in healthy controls and moderate and severe COVID-19 patients. (*E*) UMAP of CD16^bright^ and CD16^dim^ cells within pooled neutrophils from all study groups. (*F*) Median fluorescence intensity (MFI) of selected surface markers in the UMAP described in *D*. (*G*) Hierarchical clustering of the mean *z* score of the MFI of the markers in CD16^bright^ and CD16^dim^ neutrophils in healthy controls (*n* = 17) and moderate (*n* = 10) and severe (*n* = 16) COVID-19 patients. (*H*) PCA based on marker expression (MFI) on CD16^bright^ and CD16^dim^ neutrophils. *B* and *C* use Kruskall−Wallis test and two-stage Benjamini, Krieger, and Yekutieli test. Bars represent median. *P < 0.05; **P < 0.01; ****P < 0.0001.

Neutrophils have been shown to display a high degree of phenotypical heterogeneity, which has important implications for their function in several pathological contexts (30). Different neutrophil maturation states are associated with major phenotypic differences, for example, CD16 up-regulation during development (23, 30). In COVID-19 patients, particularly in those with severe disease, we observed a robust enrichment of a neutrophil subset characterized by low expression of CD16 suggesting that these were immature CD16^dim^ neutrophils (Fig. 2*A*).

A significant increase in absolute numbers was detected for both CD16^bright^ and CD16^dim^ neutrophil subsets in COVID-19 patients, which positively correlated with disease severity (Fig. 2*B*). In addition, a significant increase in the relative fraction of CD16^dim^ neutrophils was detected, indicating an overall expansion of immature neutrophils in COVID-19 patients (Fig. 2*C*).

In order to analyze the phenotypic traits of the neutrophil subsets identified above in an unsupervised way, 30,000 events in the neutrophil gate from each study participant were concatenated and then down-sampled to 0.5 million cells. By integrating the expression of 20 phenotypic markers in the UMAP analysis, we observed only modest differences when comparing neutrophils from healthy controls and COVID-19 patients, within the UMAP space (Fig. 2*D*). In addition, the CD16^bright^ and CD16^dim^ neutrophil subsets from controls and COVID-19 patients clustered separately from each other (Fig. 2*E*), indicating substantial phenotypic differences, which did not depend on the disease state.

Next, the expression of markers related to neutrophil development, migration, and activation was analyzed. The mature CD16^bright^ neutrophil subset was characterized by higher expression of CD177, CD11b, CXCR1, CXCR2, and CD62L compared to the CD16^dim^ population. On the other hand, CD16^dim^ neutrophils were characterized by higher levels of CD66b, LOX-1, and CD24, which are associated with early stages of neutrophil development (31), confirming the immature phenotype of this subset (Fig. 2 *F* and *G* and *SI Appendix*, Fig. S2).

We also observed significant up-regulation of CD66b, CD177, CD11b, CXCR4, CD147 (the receptor that binds the spike protein of SARS-CoV-2), and CD63 in mature neutrophils from COVID-19 patients compared to healthy controls, as well as significant down-regulation of CXCR2 (Fig. 2*G* and *SI Appendix*, Fig. S2*A*). No differences in the expression of markers related to either costimulatory or inhibitory functions, such as HLA-DR, CD86, and PD-L1, between healthy controls and COVID-19 patients within either neutrophil subset were detected (Fig. 2*G* and *SI Appendix*, Fig. S2).

While the presented analysis showed several phenotypic differences between healthy controls and COVID-19 patients, we were unable to detect prominent alterations between the moderate and severe patients for any of the neutrophil subsets analyzed (Fig. 2*G* and *SI Appendix*, Fig. S2). Such phenotypic similarity between the global neutrophil compartment in moderate and severe COVID-19 patients was confirmed by principal component analysis (PCA) (Fig. 2*H*).

Collectively, this analysis highlights the accumulation of immature CD16^dim^ neutrophils and the concurrent activation of the mature neutrophil fraction in peripheral blood of COVID-19 patients.

### The Abundance of Activated Neutrophil Immunotypes Correlates with Markers of Antiviral Immune Response in Moderate Patients.

**Fig. 3.**
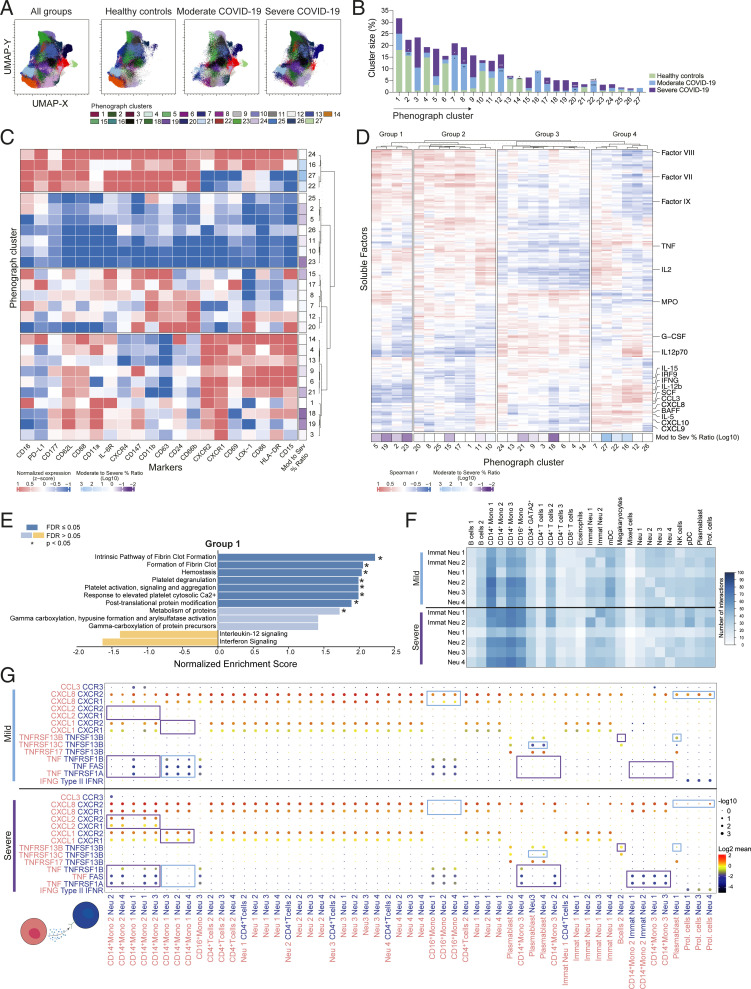
Phenograph analysis identifies activated neutrophil subsets correlating with markers of antiviral immune response in moderate COVID-19 patients. (*A*) Distribution of the 27 identified phenograph clusters (k-nearest neighbors, KNN = 235) overlaid on the UMAP projection. (*B*) Frequency of the phenograph clusters within healthy controls (*n* = 17) and moderate (*n* = 10) and severe (*n* = 16) COVID-19 patients; asterisks indicate statistically significant differences compared to healthy controls; # indicates statistically significant differences between moderate and severe patients. (*C*) Hierarchical clustering of the MFI *z* score of the markers in the phenograph clusters (*k* means = 4). Right annotation shows the log10 ratio of cluster enrichment in moderate (blue) versus severe (purple) COVID-19 patients. (*D*) Hierarchical clustering of Spearman correlation between phenograph clusters and soluble factors detected in all COVID-19 patients (*k* means = 4). Bottom annotation shows the log10 ratio of cluster enrichment in moderate (blue) versus severe (purple) COVID-19 patients. (*E*) Pathway analysis based on Spearman correlation values between soluble factors and phenograph cluster frequencies of group 1 from *D*. Only pathways displaying a normalized enrichment score of <−1 or >1 are shown. Dark blue box indicates False Discovery Rate (FDR) < 0.05. (*F*) Number of significant interactions between neutrophil subsets and other circulating immune cells from mild (*Top*) and severe (*Bottom*) COVID-19 patients as determined by applying the CellPhoneDB algorithm on a publicly available scRNAseq dataset (19). (*G*) Interaction strength (dot color) and significance (dot size) of selected ligand (pink)−receptor (blue) pairs between neutrophils and other immune subsets in mild (*Top*) and severe (*Bottom*) COVID-19 patients (19). Interactions differing between mild and severe patients are highlighted in colored boxes. Immat Neu, immature neutrophils; Mono, monocytes; Prol. Cells, proliferating cells. *B* uses Kruskall−Wallis test and two stage Benjamini, Krieger, and Yekutieli test. *P < 0.05; **P < 0.01; ***P < 0.001; ****P < 0.0001.

To further investigate whether specific neutrophil phenotypes were associated with disease progression or severity, unsupervised phenograph clustering was performed on neutrophils derived from all study participants (Fig. 3*A*). In total, 27 clusters were identified, with a varying prevalence in the three study groups (Fig. 3*B*). Hierarchical clustering based on marker expression revealed four groups of clusters (Fig. 3*C*). Specifically, the clusters that were more abundant in moderate patients (i.e., clusters 16, 22, and 27) belonged to the CD16^bright^ subset and displayed higher expression levels of CD11b, CD177, and CD66b. On the other hand, clusters that were enriched in severe COVID-19 encompassed the immature CD16^dim^ population and displayed a more heterogeneous phenotype (Fig. 3*C*). In particular, clusters 18, 19, and 23, which were more enriched in severe patients, showed lower levels of several activation markers including CD66b and CD11b (*SI Appendix*, Fig. S3*A*). Correlation of cluster frequencies in each patient with the concentration of 253 serum/plasma factors (Dataset S1) followed by hierarchical clustering highlighted specific correlation patterns associated with trends in cluster enrichment within specific disease groups (Fig. 3*D*). In particular, clusters 16, 22, and 27, which were enriched in moderate COVID-19, displayed a positive correlation with several molecules of type 1 response (CXCL9, CXCL10, and IL-15), antiviral response (interferon regulatory factor 9 [IRF9], IL-12p70, and interferon-ɣ [IFNɣ]) and adaptive immunity (B cell activating factor [BAFF] and IL-2) (Fig. 3*D*). In addition, key molecules involved in eosinophil/basophil homeostasis and recruitment (IL-5, CCL3, and stem cell factor [SCF]) were also positively correlated with these clusters. Within the clusters associated with severe patients, several members of the coagulation cascade (factors VII, VIII, and IX) and molecules associated with neutrophil maturation (i.e., granulocyte colony−stimulating factor [G-CSF] and myeloperoxidase [MPO]) were positively correlated (Fig. 3*D*). Shared patterns within cluster groups (e.g., 5, 22, 23, and 27) were also observed when separately analyzing correlations in moderate and severe COVID-19 (*SI Appendix*, Fig. S3*B* and Dataset S2), suggesting that the degree of disease severity could influence relationships between specific neutrophil immunotypes and soluble factors. Next, to gain functional insights on these associations, we performed a pathway enrichment analysis leveraging the correlation values for each of the four cluster groups with soluble factors (see *SI Appendix*, *Extended Materials and Methods*). Notably, interleukin and interferon signaling were negatively associated with groups 1 and 3, composed of clusters more abundant in severe COVID-19 patients, while pathways including platelet activation, degranulation and aggregation, hemostasis, and fibrin clot formation were significantly enriched (Fig. 1*E* and *SI Appendix*, Fig. S3*C*). This was reminiscent of recent reports showing a causative link between neutrophil extracellular traps and coagulopathies in severe COVID-19 (32–35). Conversely, coagulation and platelet activation-related pathways were down-modulated in group 4 (including clusters enriched in moderate COVID-19 patients), which showed a positive enrichment of interleukin signaling and antiviral mechanisms such as the positive regulation of ion transport (36) (*SI Appendix*, Fig. S3*C*).

Finally, to clarify the molecular mechanisms linking neutrophil subsets with the soluble factors shown in Fig. 3*D*, a receptor−ligand interaction analysis using CellPhoneDB was performed (37) on a recently published single-cell RNA sequencing (scRNAseq) dataset (19). Monocytes, dendritic cells, and a subset of CD4^+^ T cells displayed the highest number of predicted interactions with all neutrophil subsets, irrespective of the disease severity (Fig. 3*F*). When focusing on the molecules reported in Fig. 3*C*, we detected a wide set of interactions between monocyte-derived (but also CD4^+^ T cell-derived) chemokines (i.e., CCL3, CXCL1, CXCL2, and CXCL8) binding CCR3 and CXCR1/2 on neutrophil subsets, particularly evident in patients with severe COVID-19 (Fig. 3*G*) (38). Patients with mild COVID-19 displayed a marked interaction between neutrophil and plasmablasts through BAFF (TNFSF13B) and BAFF receptors (encoded by TNFRSF13C, TNFRSF13B, or TNFRSF17) (Fig. 3*G*). In patients with severe, but not mild, COVID-19, we detected the putative interaction between TNF on monocytes and different members of the TNF receptor superfamily (i.e., FAS and TNFɑR1) expressed by mature and immature neutrophils (Fig. 3*F*). Finally, neutrophils were sensitive to IFNɣ produced by proliferating T cells in both mild and severe COVID-19 patients (Fig. 3*F*).

In summary, the phenograph-guided in-depth dissection of the neutrophil subset composition allowed us to identify selected immunotypes associated either with prototypical molecules of type-1 antiviral immune response or with markers of the coagulation cascade. Moreover, our ligand−receptor interaction analysis extended these findings, highlighting the diversity of the cross-talk between neutrophils and both innate and adaptive immune cell types, with inherent alterations depending on COVID-19 severity.

### Eosinophil Activation Profile in COVID-19 Correlates with Markers of Antiviral Immune Response.

**Fig. 4.**
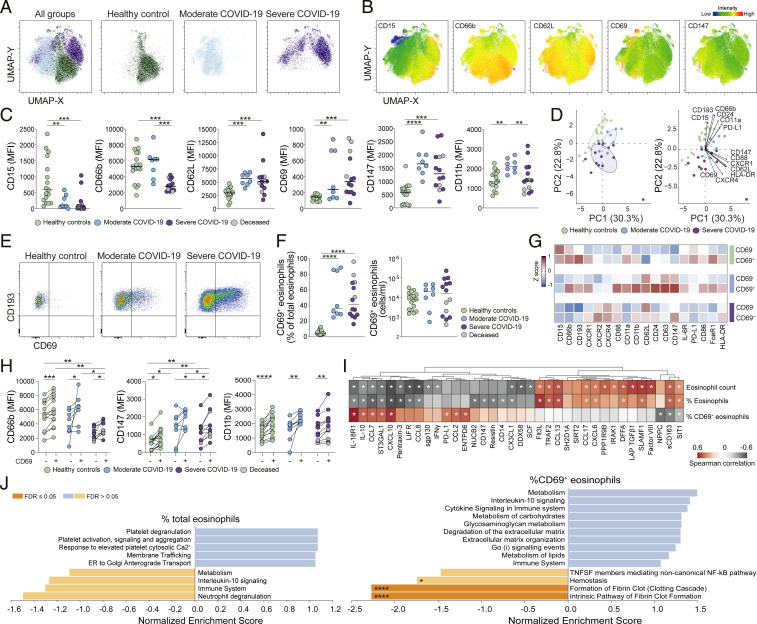
Activation of eosinophils in patients with COVID-19. (*A*) Overall and individual UMAPs on concatenated files of eosinophils in healthy controls and moderate and severe COVID-19 patients. (*B*) UMAP displaying the MFI of selected markers. (*C*) MFI of selected eosinophil markers in healthy controls (*n* = 17) and patients with moderate (*n* = 8) and severe (*n* = 14) COVID-19. Median values for each group are indicated. (*D*) PCA based on MFI expression of eosinophil markers. (*E*) CD69^+^ eosinophils from a representative healthy control and COVID-19 patients. (*F*) Frequencies and absolute counts of CD69^+^ eosinophils in healthy controls (*n* = 17) and moderate (*n* = 8) and severe (*n* = 14) COVID-19 patients. (*G*) Marker expression (*z* score) on CD69^−^ and CD69^+^ subsets. (*H*) MFI of selected markers in eosinophil subsets in healthy controls (*n* = 15) and moderate (*n* = 8) and severe (*n* = 11) COVID-19 patients. (*I*) Heatmaps demonstrating Spearman correlations (*r* < −0.4 or *r* > 0.4, *P* < 0.05) between absolute eosinophil counts/eosinophil frequency of total leukocytes/frequency of CD69^+^ eosinophils of total eosinophils and soluble factors in COVID-19 patients. (*J*) Pathway analysis based on Spearman values obtained from correlation between soluble factors and frequency of total eosinophils among total leukocytes (*Left*) or frequency of CD69^+^ eosinophils (*Right*). Only pathways displaying a normalized enrichment score of <−1 or >1 are shown. Dark orange box indicates FDR < 0.05. Statistical analysis by Kruskall−Wallis test and two-stage Benjamini, Krieger, and Yekutieli test are used in *C*, *F* and *H*, and Wilcoxon matched-pairs signed rank test is used in *H*. FDR adjusted *P* values in *C*, *F*, and *H* and *P* values < 0.05 in *J* are indicated. *P < 0.05; **P < 0.01; ***P < 0.001; ****P < 0.0001.

Through UMAP analysis integrating the expression of 21 surface markers on eosinophils, we observed that eosinophils from healthy donors clustered separately from the eosinophils of patients with COVID-19 (Fig. 4*A*), indicating significant phenotypic alterations in the eosinophil populations upon SARS-CoV-2 infection. Eosinophils from COVID-19 had decreased expression of the markers of the eosinophil lineage (CD15, CD66b, and CD193) (39) and higher expression of classical activation markers, such as CD62L, CD69, and CD147 (Fig. 4*B* and *SI Appendix*, Fig. S4*A*). Phenotypic analysis showed differences between healthy controls and COVID-19 patients, but also between patients with moderate and severe COVID-19 for some of the parameters analyzed. Patients with moderate COVID-19 expressed significantly higher levels of CD66b, CD11b, CD11a, and CD24 compared to patients with severe COVID-19 (Fig. 4*C* and *SI Appendix*, Fig. S4*B*). Notably, within the severe group, the deceased patients had the highest CD69 expression (Fig. 4*C*). PCA analysis recapitulated these differences, highlighting CD193, CD66b, CD11a, CD24, CXCR4, and CD62L as the most affected parameters in eosinophils from COVID-19 patients (Fig. 4*D*).

Furthermore, patients with COVID-19 had a significantly increased frequency of CD69^+^ eosinophils (Fig. 4 *E* and *F*). Indeed, despite the strong eosinopenia (Fig. 1*D*), the absolute counts of CD69^+^ eosinophils were not significantly altered in COVID-19 patients (Fig. 4*F*). CD69^+^ eosinophils displayed significantly higher expression of CD66b, CD147, CD11b, and CD193, compared to the corresponding CD69^−^ counterpart, confirming their activation state (Fig. 4 *G* and *H* and *SI Appendix*, Fig. S4*C*). Despite their CD69 expression, activated eosinophils from severe COVID-19 patients displayed lower CD11a, CD66b, and CD147 expression compared to CD69^+^ eosinophils from the group with moderate disease (Fig. 4 *G* and *H* and *SI Appendix*, Fig. S4*C*), suggesting a partial functional impairment of eosinophils in the more severe COVID-19 stages.

The absolute counts of eosinophils in blood correlated inversely with the peripheral levels of SCF and molecules involved in cell response to viral infection, such as DDX58 (DExD/H-Box Helicase 58, also known as RIG-I), IFNɣ, and CXCL10 (Fig. 4*I*). Conversely, the eosinophil levels in blood correlated positively with the levels of IL-1 receptor-associated kinase 1 (IRAK1); latency associated peptide-transforming growth factor β1 (LAP-TGFβ1); chemoattractants CCL13, CCL17, and CXCL6; and the coagulation factor VIII (Fig. 4*I*). The frequency of CD69^+^ eosinophils correlated positively with the levels of molecules related to IFNɣ, CCL2, CCL7, CCL8, Pentraxin-3, leukemia inhibitory factor receptor (LIFR), and PD-L1. The sCD163 correlated negatively with the frequency of CD69^+^ eosinophils but positively with the absolute counts and frequencies of eosinophils. Representative plots of the most significantly correlated parameters are shown in *SI Appendix*, Fig. S4*D*, while the most significant correlations for the moderate and severe groups separately are shown in *SI Appendix*, Fig. S4*E*.

Next, we performed a pathway enrichment analysis using soluble factors associated with eosinophil frequency (*SI Appendix*, Fig. S4*F*) or with CD69^+^ eosinophil frequency (*SI Appendix*, Fig. S4*G*). Pathways related to platelet degranulation, activation, signaling and aggregation, and response to elevated platelet cytosolic Ca2^+^ were significantly correlated with eosinophil frequency in COVID-19 patients, while neutrophil degranulation, IL-10 signaling, and metabolic pathways were negatively associated (Fig. 4*J*). On the other hand, the frequency of CD69^+^ eosinophils was positively associated with signaling of IL-10 and other cytokines, metabolic pathways, and degradation of extracellular matrix, while it was negatively associated with pathways related to fibrin clot formation, hemostasis, and TNF/NF-κB signaling.

In conclusion, our results indicate that the eosinophils in the blood of patients with COVID-19 are activated in response to SARS-CoV-2 infection and express high levels of key receptors for lung tissue infiltration, particularly in patients with moderate disease. Moreover, our findings imply the involvement of eosinophils in COVID-19 pathology through their association with platelet and neutrophil degranulation, fibrin clot formation, cytokine signaling, and degradation of extracellular matrix.

### Basophils in COVID-19 Patients Display an Activated Phenotype.

**Fig. 5.**
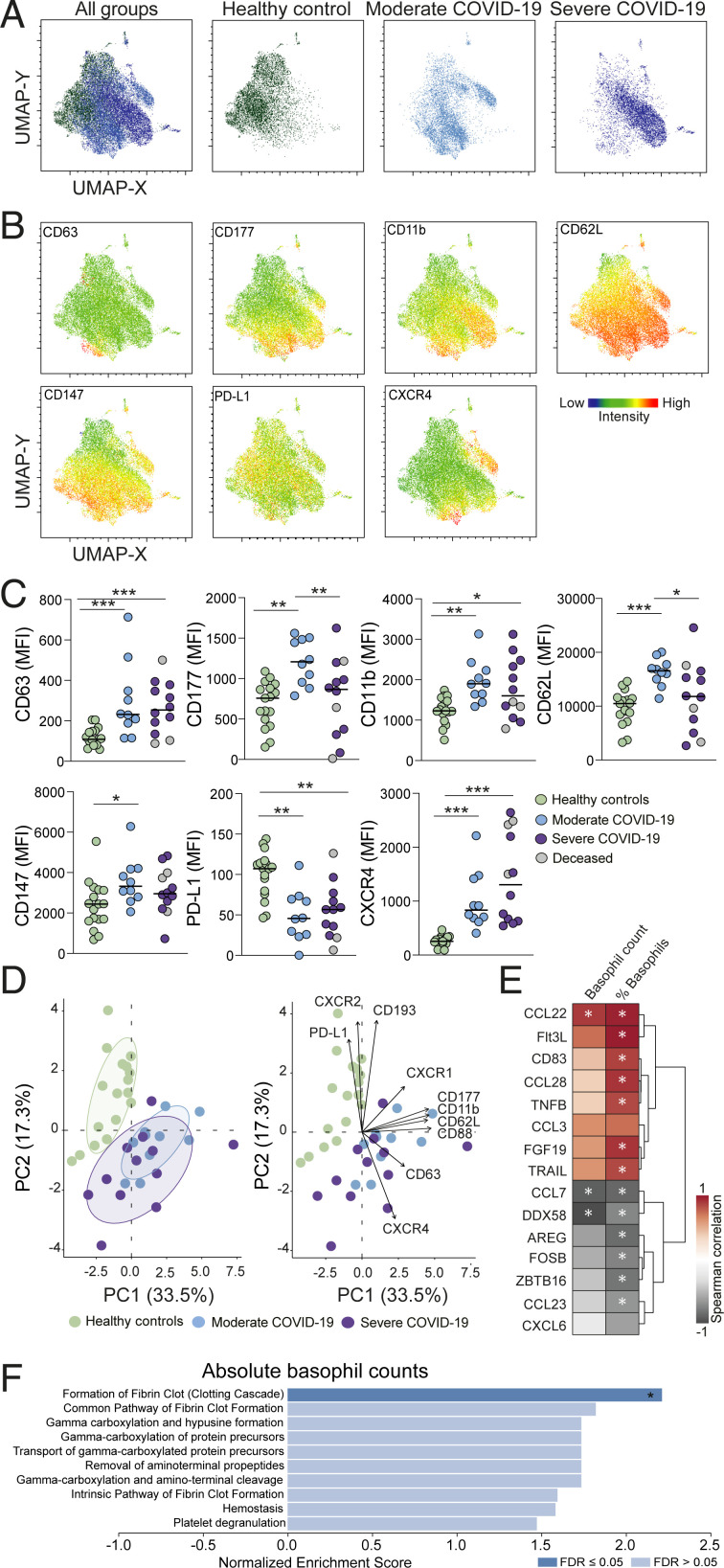
Basophils in COVID-19 patients display an activated phenotype. (*A*) UMAPs on concatenated files showing basophil overview and separate plots for healthy controls and moderate and severe COVID-19 patients. (*B*) UMAP showing the level of expression of selected markers on basophils. (*C*) MFI for selected markers on basophils in healthy controls (*n* = 17) and moderate (*n* = 10) and severe (*n* = 12) COVID-19 patients. Median values for each group are indicated. (*D*) PCA and biplot based on the MFI expression of basophil markers in healthy controls and moderate and severe COVID-19 patients. (*E*) Heatmap demonstrating Spearman correlation (*r* < −0.4 or *r* > 0.4, *P* < 0.05) between absolute basophil counts/basophil frequency of total leukocytes and basophil-associated soluble factors in COVID-19 patients. (*F*) Pathway analysis based on Spearman values obtained from correlation between soluble factors and absolute counts of basophils. Only pathways displaying a normalized enrichment score of >1 are shown. Dark blue box indicates FDR < 0.05. Significant differences between healthy controls and patient groups in *C* were evaluated with Kruskall−Wallis test and two-stage Benjamini, Krieger, and Yekutieli test. FDR adjusted *P* values in *C* and *P* values < 0.05 in *F* are indicated. *P < 0.05; **P < 0.01; ***P < 0.001.

UMAP analyses revealed distinctive basophil clusters in the healthy controls and COVID-19 patients, suggesting alterations in basophil phenotype during the disease (Fig. 5*A*). Specifically, clusters associated with COVID-19 had higher expression of markers important for the activation and recruitment of basophils, including CD11b, CD63, and CXCR4 (Fig. 5*B* and *SI Appendix*, Fig. S5*A*). Specific up-regulation of CD62L and CD147 was observed on basophils from moderate COVID-19 patients (Fig. 5*C* and *SI Appendix*, Fig. S5*B*), while PD-L1 expression was reduced in all patients compared to healthy controls. Notably, a pronounced up-regulation of CD177 was observed on basophils from COVID-19 patients, and the magnitude of its expression was comparable to the expression of CD177 in CD16^bright^ neutrophils (*SI Appendix*, Fig. S5*C*). In patients with moderate disease, CD177 expression on basophils correlated significantly with the expression of CD62L, CD147, and CD88 (*SI Appendix*, Fig. S5*D*). The differences in marker expression on basophils between healthy controls and COVID-19 patients were further confirmed by PCA, with CXCR4, CD62L, CD193, and PD-L1 among the most contributing parameters driving the separation (Fig. 5*D*). We further examined the correlation of basophils with soluble factors (Fig. 5*E* and *SI Appendix*, Fig. S5 *E* and *F*). The basophil frequency was negatively correlated with soluble DDX58, and with the transcription factors amphiregulin (AREG), FOSB, and Zinc finger, and BTB domain-containing protein 16 (ZBTB16). Similar to eosinophils, basophil frequency was also negatively correlated with CCL7. In contrast, CCL22, CCL28, and Fms-related tyrosine kinase 3 ligand (Flt3L) were positively correlated with basophil frequency, particularly in COVID-19 patients with moderate disease (Fig. 5*E* and *SI Appendix*, Fig. S5*E*). Other soluble factors that were also positively associated with basophil frequency were CD83 and tumor necrosis factor-related apoptosis-inducing ligand (TRAIL).

Pathway analysis performed using soluble factors correlated with the absolute counts of basophils (*SI Appendix*, Fig. S5*G*) revealed a significant association with pathways related to fibrin clot formation, hemostasis, and platelet degranulation, similar to what was observed in eosinophils (Fig. 5*F*).

In summary, the data show that basophils are depleted in COVID-19 patients, a pattern associated with the activation of the coagulation cascade and disease severity, and that circulating basophils from patients display an activated phenotype.

### Eosinophil Activation and Neutrophil Maturation Contribute to Predictive Models of Sequential Organ Failure Assessment Score and Respiratory Function.

**Fig. 6.**
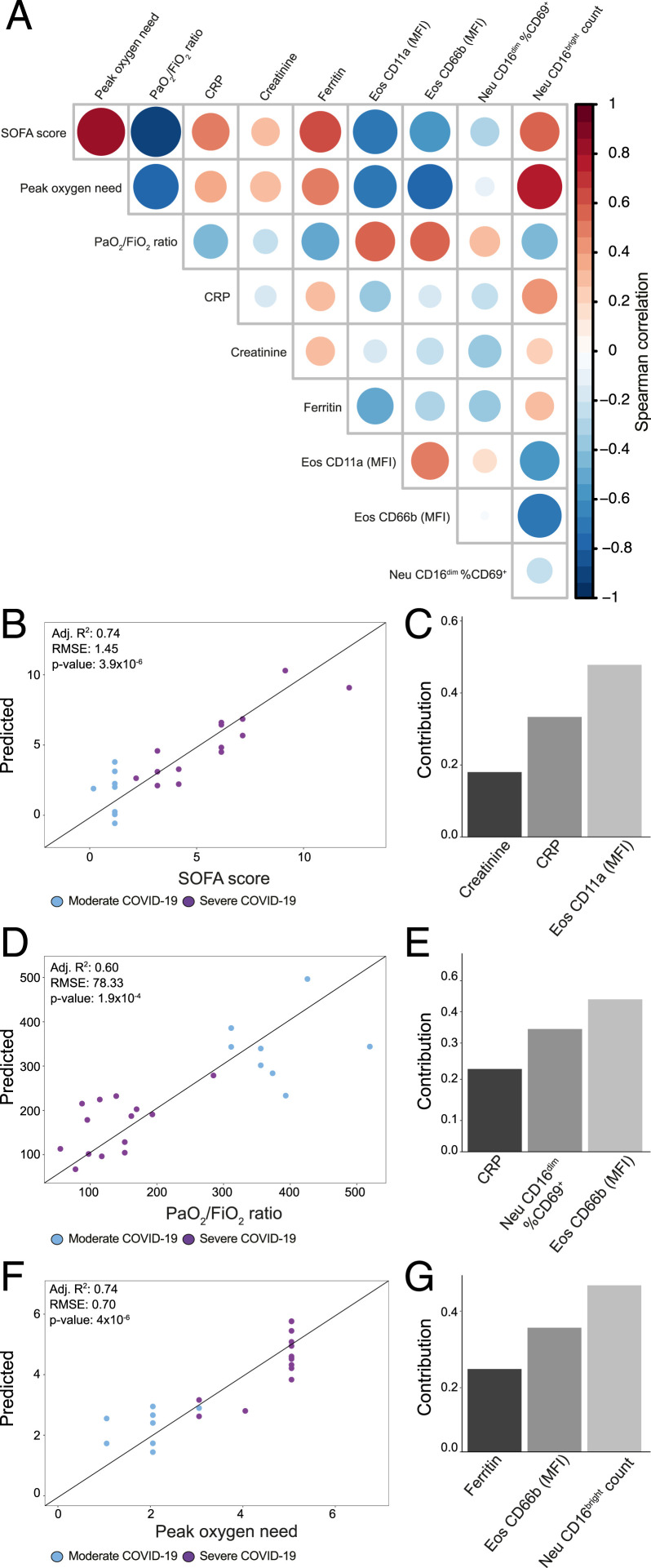
Eosinophil activation and neutrophil maturation are relevant for prediction of SOFA score and respiratory function. (*A*) Diagonal correlation matrix including predicted clinical outcomes and the explanatory variables included in the final models. (*B–G*) Multivariate linear regressions for prediction of SOFA score (*B* and *C*), PaO_2_/FiO_2_ ratio (*D* and *E*), and peak oxygen need (*F* and *G*) were modeled based on immune traits and laboratory markers. (*B*, *D*, and *F*) Scatter plots of actual values versus predicted values of the linear models. (*C*, *E*, and *G*) Individual contribution of the variables included in the final model.

Our well-defined patient cohort allowed us to compare, in detail, the phenotypic alterations in granulocyte subsets in relation to severity of COVID-19 (*SI Appendix*, Table S1). In order to elucidate the underlying relationships between immune traits and relevant clinical parameters registered during hospitalization, we performed multivariate linear regression. The models were based on a comprehensive dataset including surface marker expression levels, absolute numbers, frequencies of granulocyte subsets, available clinical information, and laboratory measurements. The final set of variables used in the predictive models were selected based on their correlation with the tested clinical outcomes and their relevance in the specific predictive models (Fig. 6*A*).

The analysis revealed that the levels of CD11a expression on eosinophils contributed to the linear model for prediction of sequential organ failure assessment (SOFA) score for patients included in our study (adjusted [Adj.] *R*^2^ = 0.74, *P* = 3.9 × 10^−6^; Fig. 6*B*). Notably, the relevance of CD11a expression on eosinophils was higher in predicting the SOFA score (explaining around 50% of the model) than the levels of two laboratory parameters, CRP and creatinine (Fig. 6*C*).

We also developed models for prediction of respiratory function, including PaO_2_/FiO_2_ ratio at baseline and maximum oxygen need during hospitalization. The linear model for PaO_2_/FiO_2_ ratio prediction (Adj. *R*^2^ = 0.60, *P* = 1.9 × 10^−4^; Fig. 6 *D* and *E*) included contributions of CD69 expression on CD16^dim^ neutrophils, of CD66b on eosinophils, and of CRP. The linear model for maximum level of oxygen administration encompassed absolute numbers of mature neutrophils, CD66b expression on eosinophils, and ferritin levels (Adj. *R*^2^ = 0.74, *P* = 4.0 × 10^−6^; Fig. 6 *F* and *G*). Notably, both models revealed a correlation between CD66b expression on eosinophils and degree of respiratory failure during COVID-19. Moreover, the combined effect of the immune parameters in both models was dominant, contributing to almost 80% of each model (Fig. 6 *C*, *E*, and *G*). Correction for relevant parameters (e.g., age, BMI, sex, comorbidities) did not significantly alter any of the linear models or the contribution of the immune cell populations to them, further strengthening the link between granulocyte activation and clinical outcome (*SI Appendix*, Table S4). To assess the value of the inclusion of granulocyte phenotypic traits in the prediction models, we developed additional models based solely on clinical parameters already available for clinicians. The resulting models showed lower performance than those including granulocyte traits (*SI Appendix*, Fig. S6 *A* and *B* and Table S5). Our results were verified by performing receiving operator characteristic analysis having severity as outcome and using the selected granulocyte traits, clinical parameters, and built linear models (*SI Appendix*, Table S6). In summary, by applying a supervised machine learning method, we identified eosinophil activation and mature neutrophil counts to be strongly correlated to SOFA score and maximum oxygen need. These immunological signatures, together with other known laboratory markers, but not alone (*SI Appendix*, Fig. S6 *C–K*), were sufficient to create predictive models.

### The Phenotype of Granulocytes Is Partly Restored in Patients Who Have Recovered from COVID-19.

**Fig. 7.**
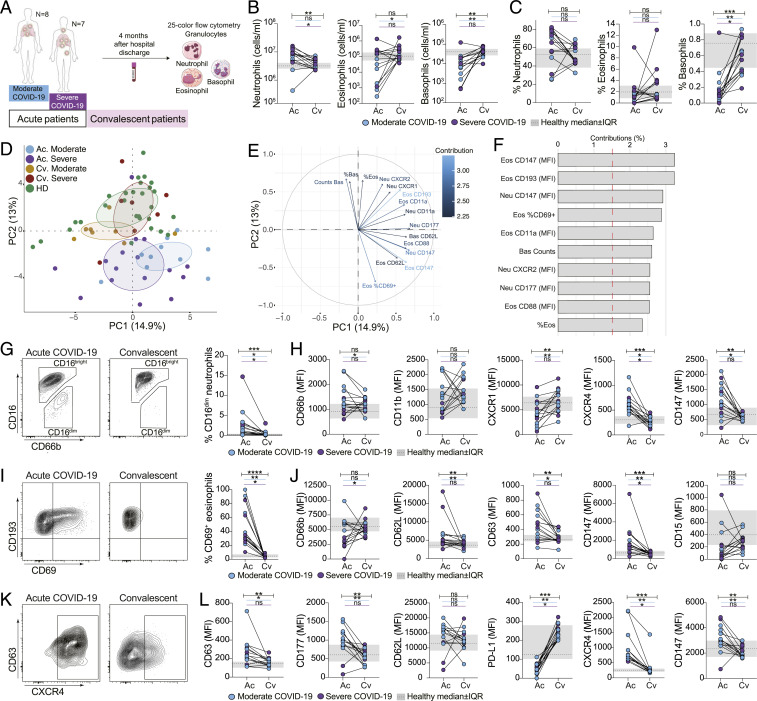
Granulocyte phenotype in convalescent patients is largely restored. (*A*) Schematic illustration of paired acute (Ac) and convalescent (Cv) patient samples included in the study. (*B*) Absolute cell counts (based on Trucount flow cytometry approach; see *Materials and Methods*) and (*C*) granulocyte frequencies over total leukocytes in paired Ac and Cv patients (*n* = 15). (*D*) PCA based on cell counts, frequencies, and normalized MFI (*z* score) of all measured markers. (*E*) Variable correlation plot. (*F*) Relative contribution of variables to PC1 and PC2. (*G*) CD16^dim^ neutrophil frequencies in paired Ac and Cv patient samples (*n* = 15). (*H*) Expression of selected neutrophil markers in paired Ac and Cv patients (*n* = 15). (*I*) CD69 eosinophil expression in paired Ac and Cv patients (*n* = 15). (*J*) Expression of selected eosinophil markers in paired Ac and Cv patient samples (*n* = 15). (*K*) CXCR4 basophil expression in Ac and Cv patients. (*L*) Expression of selected basophil markers in paired Ac and Cv patients (*n* = 15). Wilcoxon matched-pairs rank test was used; ns, not significant; *P < 0.05; **P < 0.01; ***P < 0.001; ****P < 0.0001. In *B*, *C*, *H*, *J*, and *L*, healthy controls (*n* = 28, median ± interquartile, IQR) ranges are shown in gray. Bars indicate statistical significance considering all sampled patients (black) or only moderate (blue) or only severe (purple) patients.

To determine whether the phenotypical alterations observed within the granulocyte compartment during acute COVID-19 were recovered after viral clearance, whole blood samples were collected from the same moderate (*n* = 8) or severe (*n* = 7) patients approximately 4 mo (median = 136 d, range = 89 d to 153 d) after hospital discharge (Fig. 7*A*). Convalescent samples displayed normalized cell counts for neutrophils, eosinophils, and basophils, as well as NLR, compared to the samples collected during acute viral infection (Fig. 7*B* and *SI Appendix*, Fig. S7*A*), while the relative abundance of the granulocyte subsets over total leukocytes was only partially restored (Fig. 7*C*), suggesting that circulating nongranulocytic immune cells might also still be quantitatively affected. Nevertheless, both absolute numbers and frequencies of granulocyte subsets in convalescent patients were very similar to those observed in healthy controls (Fig. 7 *B* and *C*). Additionally, PCA highlighted that convalescent patients were markedly more similar to healthy controls than to acute patients (Fig. 7*D*). In particular, CD69 and CD193 expression on eosinophils, as well as CD147 expression on neutrophils and eosinophils, as well as basophil counts were among the variables contributing most to the observed clustering (Fig. 7 *E* and *F* and *SI Appendix*, Fig. S7*B*).

Immature CD16^dim^ neutrophils, elevated in acute COVID-19, were almost absent in convalescence (Fig. 7*G*). Neutrophils from convalescent patients showed normalized levels of CXCR1, CXCR4, and CD147 compared to acute infection (Fig. 7*H* and *SI Appendix*, Fig. S7*C*). Eosinophil activation was also reduced in convalescent patients, as shown by the normalized expression of CD69, CD62L, CD63, and CD147, as well as by the increased PD-L1 expression (Fig. 7 *I* and *J* and *SI Appendix*, Fig. S7*D*). However, the expression of CD66b, CD15, and CD193 on eosinophils was not completely recovered in convalescent samples when compared to healthy controls (Fig. 7*J* and *SI Appendix*, Fig. S7*D*), suggesting that a partial phenotypic impairment might still be present in the eosinophil population. Similarly, basophils in the convalescent samples showed an overall recovery of several phenotypic traits altered in acute COVID-19. In particular, the expression levels of CD11b, CD63, CXCR4, and PD-L1 (Fig. 7 *K* and *L* and *SI Appendix*, Fig. S7*E*) were in line with the levels found in healthy controls.

## Discussion

The reported clinical relevance of the inflammatory state in the more severe forms of COVID-19 has raised an unprecedented awareness of the potential detrimental role of polymorphonuclear cells in acute viral infections (19). However, a comprehensive phenotypic description performed at the protein level on circulating granulocytes in SARS-CoV-2−infected patients is limited. In this report, we show that immature CD16^dim^ neutrophils increase in number and frequency in peripheral blood of COVID-19 patients and confirm that general neutrophilia can be considered a hallmark of severe COVID-19. However, in contrast to the tenet linking neutrophil activation with more-severe clinical conditions, our approach based on high-dimensional flow cytometry was able to identify activated neutrophil immunotypes detected predominantly in moderate patients showing a better clinical outcome. On the same lines, our UMAP analysis indicated a marked phenotypic difference between granulocytes from moderate COVID-19 and healthy controls, which was less evident for granulocytes from severe COVID-19. Furthermore, we extended our analysis to other granulocyte subsets, reporting the substantial ablation of both eosinophils and basophils in peripheral blood of COVID-19 patients. Such depletion could possibly result from two nonmutually exclusive phenomena: first, both cell types might be recruited to inflamed tissues, and, in particular, to the lung. This hypothesis is supported by the relevant expression changes of several adhesion/migration molecules (e.g., CD62L, CD11a/b, and CXCR4) on both eosinophils and basophils. Moreover, concentrations of key soluble factors involved in the recruitment of both granulocyte subsets (e.g., CCL13, CCL17, CCL22, and CCL28) (40) correlated with their levels in circulation. A second explanation for the detected basopenia/eosinopenia in favor of an expanded neutrophil compartment might imply the reprogramming of the granulocyte precursor in the bone marrow which typically occurs during emergency hematopoiesis (41).

The increase of immature neutrophil counts in circulation has been observed in previous studies investigating a variety of viral infections, including HIV, dengue, and SARS-CoV-2 (18, 19, 42–45), and has been associated with emergency granulopoiesis. Recent studies identified high CD63 messenger RNA levels as part of an immature proneutrophil/preneutrophil signature (31), which was found to be significantly enriched in COVID-19 patients (19). In this regard, we found a robust CD63 up-regulation on CD16^dim^ neutrophils and a further increased expression in COVID-19 patients. Both moderate and severe COVID-19 patients were characterized by the progressive expansion of CD16^dim^ neutrophils, which also expressed lower levels of CD177, CD11b, and CD62L and higher levels of CD66b and LOX-1, a phenotype compatible with neutrophil immaturity (31).

Our in-depth phenograph-guided analysis of the neutrophil compartment revealed the enrichment of activated neutrophil immunotypes in moderate COVID-19 patients, associated with higher circulating levels of molecules involved in the IFN-mediated antiviral response (i.e., IRF9, IL-12b, IFNɣ). Neutrophils are known to be highly responsive to type I IFN in different contexts (46, 47), and recent studies described the existence of an IFN-responsive, developmentally distinct, neutrophil subset (48). Supporting the relevance of neutrophil immunomodulation in COVID-19, we describe several putative interaction axes linking neutrophils with both innate and adaptive components of the immune response, particularly monocytes and plasmablasts. In this regard, a “B cell helper” neutrophil subset has been previously reported to promote B cell maturation and plasmablast differentiation in a BAFF-dependent manner (49). Intriguingly, we observed a positive correlation between type 1 inflammatory mediators (e.g., IFNɣ and CXCL10) and eosinophil activation, suggesting that, particularly in moderately affected patients, part of the granulocyte compartment could be actively participating in the efficient viral clearance, similarly to what can occur upon influenza infection (50, 51). Interestingly, CD62L expression on eosinophils can be triggered by IFNɣ, and CD62L-expressing eosinophils have been suggested to contribute to dysregulated inflammation and ARDS in acute COVID-19 (24, 52).

Eosinophils have been reported to express receptors that allow the recognition and orchestration of antiviral responses to respiratory viruses (51, 53, 54). Eosinophil-associated lung pathology has been reported in other viral infections and in SARS-CoV-1 vaccination studies (summarized in ref. 55). On the other hand, the role of human basophils in viral infections is poorly understood and mainly focused on basophil response to HIV (56, 57). CXCR4 is one of the most highly expressed basophil receptors in COVID-19 patients in our cohort and might be implicated in basophil transendothelial migration (57). CD63 expression on basophils can be induced by cross-linking of CD62L and CD11b, among other stimuli (58). Therefore, the up-regulation of CD62L, CD63, CD11b, and CXCR4 on basophils observed during the acute phase of COVID-19 and their normalization after viral clearance might imply a role of this phenotype in COVID-19 pathophysiology.

In contrast to the neutrophil phenotypes observed in moderate COVID-19, the neutrophil clusters enriched in patients with severe disease correlated with several coagulation factors and with molecules involved in neutrophil maturation (G-CSF and MPO), but not with any of the screened antiviral response-related molecules. This suggests an impaired neutrophil response during the more severe phases of the disease. Notably, we did not detect evident signs of immune-suppressive activity (e.g., PD-L1 expression) in our phenotypic analysis, and direct functional evidence will be crucial to assess whether increased suppressive functions are triggered in neutrophils in severe COVID-19 patients. In addition, despite their CD69 expression, activated eosinophils in severe COVID-19 patients displayed lower levels of CD11a, CD63, and CD66b compared to the CD69^+^ eosinophils from patients with moderate COVID-19. This phenotype suggests that they might be activated eosinophils that have recently degranulated (39, 59). Overall, our analyses showed that the robust activation observed in the granulocyte compartment in moderate COVID-19 declines in the more severe stages of the disease.

Combination of eosinopenia with elevated CRP could effectively triage suspected patients with COVID-19 from other patients with fever (60). A relevant finding emerging from our study is that selected granulocytic phenotypical traits could, in concert with other known laboratory markers such as CRP or creatinine, predict, to a significant extent, key clinical outcomes, including respiratory functionality and SOFA score. Although this finding needs to be taken with caution considering the limited size of our patient cohort, we provide the proof of principle that the combination of immunological, and specifically granulocyte-related, measurements with standard clinical data could be used in generating algorithms for patient classification and, thus, tailored therapeutic regimens. Notably, the immunological parameters were predominant in driving the prediction models in comparison to standard laboratory measurements. On the same lines, we connected clinical manifestations, such as multiorgan failure and pulmonary function, with specific phenotypes, primarily the eosinophil activation markers CD11a, CD66b, and CD69. Future studies will be important to assess and validate whether there is a mechanistic link between granulocyte activation and specific clinical features of COVID-19 patients.

Finally, by taking advantage of paired longitudinal sampling, we report the almost complete phenotypic recovery upon viral clearance of the phenotypic alterations observed in granulocytes from acute SARS-CoV-2−infected patients. Our findings are in agreement with previous studies showing the replenishment of the eosinophil and basophil pool and the normalization of neutrophil numbers in circulation (24, 61). In addition, the current study provides in depth characterization of the eosinophil and basophil profile in patients with COVID-19 and their phenotypic alterations linked to disease. This is emphasized by the finding that 6 out of the 10 most significant immunological parameters that drive the separate clustering of patients with acute COVID-19 from matched convalescent samples or healthy controls are eosinophil traits. However, the expression of a set of markers (e.g., CD15, CD66b) was not completely recovered in convalescent samples when compared to noninfected healthy controls, suggesting a partial remaining phenotypic impairment in the granulocyte population in convalescent samples, at least 4 mo after hospital discharge.

The inference of mechanisms driving COVID-19 immunopathology from the analysis of circulating granulocytes might have limitations when considering the pulmonary damage displayed in COVID-19 patients. However, the activation of the coagulation cascade was significantly correlated with both quantitative and qualitative changes in all granulocyte populations analyzed in severe COVID-19, supporting the clinical relevance of our findings. In addition, several studies have shown that the combined use of peripheral immune signatures, soluble factors, and patient metadata retain the capacity to predict clinical outcome to a notable extent (6, 61). Moreover, accumulating evidence indicates that other tissues (e.g., kidney, gut, brain) are affected by COVID-19−related immune alterations, thus underscoring the systemic nature of this disease and emphasizing the relevance of studying alterations in peripheral immune cells (62). Our findings highlight the significant alterations of granulocyte subpopulations in frequency and function in the blood of patients with COVID-19. Moreover, our data indicate the potential contribution of granulocytes to SARS-CoV-2 immunopathology and point toward the combined use of granulocyte-related immunological parameters and basic clinical laboratory tests as better prognostic biomarkers of disease severity and disease course.

## Materials and Methods

### Study Cohort.

SARS-CoV-2−infected patients with COVID-19 admitted at the intensive care or high-dependency unit (*n* = 16, severe COVID-19) or the infectious disease clinic (*n* = 10, moderate COVID-19) at Karolinska University Hospital, Stockholm, Sweden, were recruited to this study. Serum and (ethylenedinitrilo)tetraacetic acid (EDTA) blood samples were collected and analyzed. Inclusion and exclusion criteria for patient enrollment and collected clinical information and laboratory values of the patients are provided in *SI Appendix*, Tables S1–S3. Convalescent serum and EDTA samples were later collected from 15 patients (8 and 7, from the moderate and severe groups, respectively) ∼4 mo after hospital discharge (median = 136 d, range = 89 d to 153 d). As controls, serum and blood samples from age- and sex-matched SARS-CoV-2 IgG seronegative healthy volunteers (*SI Appendix*, Table S1) were collected on the same days as for the acute COVID-19 (*n* = 17) and convalescent (*n* = 11) patients. The iFlash-SARS-CoV-2 IgG (Yhlo Biotech), a paramagnetic particle-based chemiluminescent immunoassay was used to determine the IgG antibodies against SARS-CoV-2 nucleocapsid and spike protein (96.3% specificity and 97.3% sensitivity). The results were reported as either nonreactive (negative) < 10.0 AU/mL or reactive (positive) > 10.0 AU. All samples were processed using the same standardized operating procedures. Informed consent was obtained from all study participants, and the study was approved by the regional Ethics Committee in Stockholm, Sweden, and performed in accordance with the Declaration of Helsinki.

### Absolute Counts of Leukocytes in Peripheral Blood.

Absolute counts of peripheral blood leukocytes were determined for all study participants using BD Trucount tubes, six-color TBNK Reagent and, in addition, CD123 BUV395 (BD Biosciences), CD15 PB, CD193 BV605, and HLA-DR BV785 (Biolegend), and CD14 PE-Cy5 (eBioscience) according to the manufacturer’s instructions. Samples were fixed in 1× BD fluorescence-activated cell sorter (FACS) lysing solution (BD Biosciences) for 2 h and acquired on a BD FACSymphony A5 instrument, equipped with ultraviolet (355nm), violet (405 nm), blue (488 nm), yellow/green (561 nm), and red (637 nm) lasers. For absolute cell count calculations, the number of events for populations of interest was divided by the number of bead events and multiplied by the BD Trucount bead count.

### Cell Preparation and Staining for Multicolor Flow Cytometry.

For flow cytometric analysis of polymorphonuclear leukocytes, EDTA whole blood samples from all patients and controls were stained with fluorescently labeled antibodies (*SI Appendix*, Table S7). In brief, plasma from whole blood was removed by centrifugation, and leukocytes were washed with FACS buffer (phosphate-buffered saline, 5% fetal calf serum, 0.05 mM EDTA), followed by a 15-min incubation with a mixture containing Fc-block (Miltenyi), fluorescently-labeled antibodies for extracellular staining, and the fixable LIVE/DEAD Yellow Dead Cell Stain Kit (Life Technologies). The cells were then washed twice with FACS buffer and fixed with BD Cytofix/Cytoperm (BD Biosciences) for 15 min. Subsequently, the cells from both patients and controls were washed twice with 1× Perm/wash solution (BD Biosciences) and incubated for two additional hours in ultrapure, methanol-free, 1% formaldehyde (Polyscience) to ensure SARS-CoV-2 inactivation. Further washes with 1× Perm/wash solution ensured that all lysed red blood cells (RBC) due to Cytofix/Cytoperm incubation were removed from the samples. Due to heavy RBC coagulation in several COVID-19 patient samples, an additional RBC lysis with Cytofix/Cytoperm for 10 min was performed followed by washes with 1× Perm/wash solution. The cells were finally resuspended in FACS buffer, and samples were acquired on a BD FACSymphony equipped with five lasers (BD Biosciences).

## Supplementary Material

Supplementary File

Supplementary File

Supplementary File

## Data Availability

Curated flow cytometry data will be made available for exploration via the KI/K COVID-19 Immune Atlas, https://covid19cellatlas.com/. All other data needed to evaluate the conclusions of the paper are presented in the paper or in *SI Appendix*. Raw flow cytometry data (fcs files) can be made available upon reasonable request from the corresponding author (M.L.).

## References

[1] W. J. Guan ., Clinical characteristics of coronavirus disease 2019 in China. N. Engl. J. Med. 382, 1708–1720 (2020). 32109013 10.1056/NEJMoa2002032 PMC7092819

[2] Z. Wu, J. M. McGoogan, Characteristics of and important lessons from the coronavirus disease 2019 (COVID-19) outbreak in China: Summary of a report of 72314 cases from the Chinese Center for Disease Control and Prevention. JAMA 323, 1239–1242 (2020). 32091533 10.1001/jama.2020.2648

[3] P. Mehta ., COVID-19: Consider cytokine storm syndromes and immunosuppression. Lancet 395, 1033–1034 (2020). 32192578 10.1016/S0140-6736(20)30628-0 PMC7270045

[4] Y. Yang ., Plasma IP-10 and MCP-3 levels are highly associated with disease severity and predict the progression of COVID-19. J. Allergy Clin. Immunol. 146, 119–127.e4 (2020). 32360286 10.1016/j.jaci.2020.04.027 PMC7189843

[5] D. Blanco-Melo ., Imbalanced host response to SARS-CoV-2 drives development of COVID-19. Cell 181, 1036–1045.e9 (2020). 32416070 10.1016/j.cell.2020.04.026 PMC7227586

[6] C. Lucas ., Longitudinal analyses reveal immunological misfiring in severe COVID-19. Nature 584, 463–469 (2020). 32717743 10.1038/s41586-020-2588-y PMC7477538

[7] A. Mantovani, M. A. Cassatella, C. Costantini, S. Jaillon, Neutrophils in the activation and regulation of innate and adaptive immunity. Nat. Rev. Immunol. 11, 519–531 (2011). 21785456 10.1038/nri3024

[8] J. Liu ., Longitudinal characteristics of lymphocyte responses and cytokine profiles in the peripheral blood of SARS-CoV-2 infected patients. EBioMedicine 55, 102763 (2020). 32361250 10.1016/j.ebiom.2020.102763 PMC7165294

[9] R. Channappanavar ., Sex-based differences in susceptibility to severe acute respiratory syndrome coronavirus infection. J. Immunol. 198, 4046–4053 (2017). 28373583 10.4049/jimmunol.1601896 PMC5450662

[10] S. H. Alfaraj ., Clinical predictors of mortality of Middle East Respiratory Syndrome Coronavirus (MERS-CoV) infection: A cohort study. Travel Med. Infect. Dis. 29, 48–50 (2019). 30872071 10.1016/j.tmaid.2019.03.004 PMC7110962

[11] J. J. Lee ., Human versus mouse eosinophils: “That which we call an eosinophil, by any other name would stain as red.” J. Allergy Clin. Immunol. 130, 572–584 (2012). 22935586 10.1016/j.jaci.2012.07.025 PMC3496419

[12] J. V. Camp, C. B. Jonsson, A role for neutrophils in viral respiratory disease. Front. Immunol. 8, 550 (2017). 28553293 10.3389/fimmu.2017.00550 PMC5427094

[13] T. A. Miura, K. V. Holmes, Host-pathogen interactions during coronavirus infection of primary alveolar epithelial cells. J. Leukoc. Biol. 86, 1145–1151 (2009). 19638499 10.1189/jlb.0209078 PMC2774885

[14] H. Karasuyama, K. Miyake, S. Yoshikawa, Y. Yamanishi, Multifaceted roles of basophils in health and disease. J. Allergy Clin. Immunol. 142, 370–380 (2018). 29247714 10.1016/j.jaci.2017.10.042

[15] H. F. Rosenberg, K. D. Dyer, J. B. Domachowske, Eosinophils and their interactions with respiratory virus pathogens. Immunol. Res. 43, 128–137 (2009). 18818885 10.1007/s12026-008-8058-5 PMC2777531

[16] M. Liao ., Single-cell landscape of bronchoalveolar immune cells in patients with COVID-19. Nat. Med. 26, 842–844 (2020). 32398875 10.1038/s41591-020-0901-9

[17] R. L. Chua ., COVID-19 severity correlates with airway epithelium-immune cell interactions identified by single-cell analysis. Nat. Biotechnol. 38, 970–979 (2020). 32591762 10.1038/s41587-020-0602-4

[18] A. J. Wilk ., A single-cell atlas of the peripheral immune response in patients with severe COVID-19. Nat. Med. 26, 1070–1076 (2020). 32514174 10.1038/s41591-020-0944-y PMC7382903

[19] J. Schulte-Schrepping ., Severe COVID-19 is marked by a dysregulated myeloid cell compartment. Cell 182, 1419–1440.e23 (2020). 32810438 10.1016/j.cell.2020.08.001 PMC7405822

[20] C. Agrati ., Expansion of myeloid-derived suppressor cells in patients with severe coronavirus disease (COVID-19). Cell Death Differ. 27, 3196–3207 (2020). 32514047 10.1038/s41418-020-0572-6 PMC7278239

[21] J. Hadjadj ., Impaired type I interferon activity and inflammatory responses in severe COVID-19 patients. Science 369, 718–724 (2020). 32661059 10.1126/science.abc6027 PMC7402632

[22] C. Silvestre-Roig, Z. G. Fridlender, M. Glogauer, P. Scapini, Neutrophil diversity in health and disease. Trends Immunol. 40, 565–583 (2019). 31160207 10.1016/j.it.2019.04.012 PMC7185435

[23] S. Jaillon ., Neutrophil diversity and plasticity in tumour progression and therapy. Nat. Rev. Cancer 20, 485–503 (2020). 32694624 10.1038/s41568-020-0281-y

[24] L. Rodriguez ., Systems-level immunomonitoring from acute to recovery phase of severe COVID-19. Cell Rep Med 1, 100078 (2020). 32838342 10.1016/j.xcrm.2020.100078 PMC7405891

[25] T. Sekine ., Robust T cell immunity in convalescent individuals with asymptomatic or mild COVID-19. Cell 183, 158–168.e14 (2020). 32979941 10.1016/j.cell.2020.08.017 PMC7427556

[26] C. Maucourant ., Natural killer cell immunotypes related to COVID-19 disease severity. Sci. Immunol. 5, eabd6832 (2020). 32826343 10.1126/sciimmunol.abd6832 PMC7665314

[27] T. Parrot ., MAIT cell activation and dynamics associated with COVID-19 disease severity. Sci. Immunol. 5, eabe1670 (2020). 32989174 10.1126/sciimmunol.abe1670 PMC7857393

[28] M. García ., Innate lymphoid cell composition associates with COVID-19 disease severity. Clin. Transl. Immunology 9, e1224 (2020). 33343897 10.1002/cti2.1224 PMC7734472

[29] J. Liu ., Neutrophil-to-lymphocyte ratio predicts critical illness patients with 2019 coronavirus disease in the early stage. J. Transl. Med. 18, 206 (2020). 32434518 10.1186/s12967-020-02374-0 PMC7237880

[30] L. G. Ng, R. Ostuni, A. Hidalgo, Heterogeneity of neutrophils. Nat. Rev. Immunol. 19, 255–265 (2019). 30816340 10.1038/s41577-019-0141-8

[31] I. Kwok ., Combinatorial single-cell analyses of granulocyte-monocyte progenitor heterogeneity reveals an early uni-potent neutrophil progenitor. Immunity 53, 303–318.e5 (2020). 32579887 10.1016/j.immuni.2020.06.005

[32] E. A. Middleton ., Neutrophil extracellular traps contribute to immunothrombosis in COVID-19 acute respiratory distress syndrome. Blood 136, 1169–1179 (2020). 32597954 10.1182/blood.2020007008 PMC7472714

[33] L. Nicolai ., Immunothrombotic dysregulation in COVID-19 pneumonia is associated with respiratory failure and coagulopathy. Circulation 142, 1176–1189 (2020). 32755393 10.1161/CIRCULATIONAHA.120.048488 PMC7497892

[34] M. Leppkes ., Vascular occlusion by neutrophil extracellular traps in COVID-19. EBioMedicine 58, 102925 (2020). 32745993 10.1016/j.ebiom.2020.102925 PMC7397705

[35] B. J. Barnes ., Targeting potential drivers of COVID-19: Neutrophil extracellular traps. J. Exp. Med. 217, e20200652 (2020). 32302401 10.1084/jem.20200652 PMC7161085

[36] J. D. Londino ., Influenza virus infection alters ion channel function of airway and alveolar cells: Mechanisms and physiological sequelae. Am. J. Physiol. Lung Cell. Mol. Physiol. 313, L845–L858 (2017). 28775098 10.1152/ajplung.00244.2017 PMC5792181

[37] M. Efremova, M. Vento-Tormo, S. A. Teichmann, R. Vento-Tormo, CellPhoneDB: Inferring cell-cell communication from combined expression of multi-subunit ligand-receptor complexes. Nat. Protoc. 15, 1484–1506 (2020). 32103204 10.1038/s41596-020-0292-x

[38] M. Metzemaekers ., Kinetics of peripheral blood neutrophils in severe coronavirus disease 2019. Clin. Transl. Immunology 10, e1271 (2021). 33968405 10.1002/cti2.1271 PMC8082714

[39] J. Yoon, A. Terada, H. Kita, CD66b regulates adhesion and activation of human eosinophils. J. Immunol. 179, 8454–8462 (2007). 18056392 10.4049/jimmunol.179.12.8454

[40] S. Yi ., Eosinophil recruitment is dynamically regulated by interplay among lung dendritic cell subsets after allergen challenge. Nat. Commun. 9, 3879 (2018). 30250029 10.1038/s41467-018-06316-9 PMC6155158

[41] M. G. Manz, S. Boettcher, Emergency granulopoiesis. Nat. Rev. Immunol. 14, 302–314 (2014). 24751955 10.1038/nri3660

[42] P. L. Guo ., The clinical significance of myeloid-derived suppressor cells in dengue fever patients. BMC Infect. Dis. 19, 926 (2019). 31675923 10.1186/s12879-019-4574-2 PMC6824033

[43] A. Silvin ., Elevated calprotectin and abnormal myeloid cell subsets discriminate severe from mild COVID-19. Cell 182, 1401–1418.e18 (2020). 32810439 10.1016/j.cell.2020.08.002 PMC7405878

[44] N. L. Bowers ., Immune suppression by neutrophils in HIV-1 infection: Role of PD-L1/PD-1 pathway. PLoS Pathog. 10, e1003993 (2014). 24626392 10.1371/journal.ppat.1003993 PMC3953441

[45] G. Carissimo ., Whole blood immunophenotyping uncovers immature neutrophil-to-VD2 T-cell ratio as an early marker for severe COVID-19. Nat. Commun. 11, 5243 (2020). 33067472 10.1038/s41467-020-19080-6 PMC7568554

[46] J. Jablonska, S. Leschner, K. Westphal, S. Lienenklaus, S. Weiss, Neutrophils responsive to endogenous IFN-beta regulate tumor angiogenesis and growth in a mouse tumor model. J. Clin. Invest. 120, 1151–1164 (2010). 20237412 10.1172/JCI37223 PMC2846036

[47] P. Mistry ., Transcriptomic, epigenetic, and functional analyses implicate neutrophil diversity in the pathogenesis of systemic lupus erythematosus. Proc. Natl. Acad. Sci. U.S.A. 116, 25222–25228 (2019). 31754025 10.1073/pnas.1908576116 PMC6911190

[48] X. Xie ., Single-cell transcriptome profiling reveals neutrophil heterogeneity in homeostasis and infection. Nat. Immunol. 21, 1119–1133 (2020). 32719519 10.1038/s41590-020-0736-z PMC7442692

[49] I. Puga ., B cell-helper neutrophils stimulate the diversification and production of immunoglobulin in the marginal zone of the spleen. Nat. Immunol. 13, 170–180 (2011). 22197976 10.1038/ni.2194 PMC3262910

[50] B. M. Tang ., Neutrophils-related host factors associated with severe disease and fatality in patients with influenza infection. Nat. Commun. 10, 3422 (2019). 31366921 10.1038/s41467-019-11249-y PMC6668409

[51] G. F. Rimmelzwaan, M. M. Baars, P. de Lijster, R. A. Fouchier, A. D. Osterhaus, Inhibition of influenza virus replication by nitric oxide. J. Virol. 73, 8880–8883 (1999). 10482647 10.1128/jvi.73.10.8880-8883.1999 PMC112914

[52] C. Mesnil ., Lung-resident eosinophils represent a distinct regulatory eosinophil subset. J. Clin. Invest. 126, 3279–3295 (2016). 27548519 10.1172/JCI85664 PMC5004964

[53] A. S. Flores-Torres, M. C. Salinas-Carmona, E. Salinas, A. G. Rosas-Taraco, Eosinophils and respiratory viruses. Viral Immunol. 32, 198–207 (2019). 31140942 10.1089/vim.2018.0150

[54] M. G. Drake ., Human and mouse eosinophils have antiviral activity against parainfluenza virus. Am. J. Respir. Cell Mol. Biol. 55, 387–394 (2016). 27049514 10.1165/rcmb.2015-0405OC PMC5023029

[55] A. W. Lindsley, J. T. Schwartz, M. E. Rothenberg, Eosinophil responses during COVID-19 infections and coronavirus vaccination. J. Allergy Clin. Immunol. 146, 1–7 (2020). 32344056 10.1016/j.jaci.2020.04.021 PMC7194727

[56] A. P. Jiang ., Human blood-circulating basophils capture HIV-1 and mediate viral trans-infection of CD4+ T cells. J. Virol. 89, 8050–8062 (2015). 26018157 10.1128/JVI.01021-15 PMC4505656

[57] F. W. Rossi ., HIV-1 Nef promotes migration and chemokine synthesis of human basophils and mast cells through the interaction with CXCR4. Clin. Mol. Allergy 14, 15 (2016). 27822141 10.1186/s12948-016-0052-1 PMC5088669

[58] F. Kalm, L. Mansouri, A. Russom, J. Lundahl, A. Nopp, Adhesion molecule cross-linking and cytokine exposure modulate IgE- and non-IgE-dependent basophil activation. Immunology 162, 92–104 (2021). 32955733 10.1111/imm.13268 PMC7730031

[59] H. F. Rosenberg, K. D. Dyer, P. S. Foster, Eosinophils: Changing perspectives in health and disease. Nat. Rev. Immunol. 13, 9–22 (2013). 23154224 10.1038/nri3341 PMC4357492

[60] Q. Li ., Eosinopenia and elevated C-reactive protein facilitate triage of COVID-19 patients in fever clinic: A retrospective case-control study. EClinicalMedicine 23, 100375 (2020). 32368728 10.1016/j.eclinm.2020.100375 PMC7196382

[61] A. G. Laing ., A dynamic COVID-19 immune signature includes associations with poor prognosis. Nat. Med. 26, 1623–1635 (2020). 32807934 10.1038/s41591-020-1038-6

[62] N. Vabret ., Immunology of COVID-19: Current state of the science. Immunity 52, 910–941 (2020). 32505227 10.1016/j.immuni.2020.05.002 PMC7200337

