# Supplementary material for: High-dimensional profiling reveals phenotypic heterogeneity and disease-specific alterations of granulocytes in COVID-19
Source: Proc Natl Acad Sci U S A. 2021 Sep 21;118(40):e2109123118. doi: 10.1073/pnas.2109123118 (PMC8501786; doi:10.1073/pnas.2109123118)
Supplement: Supplementary File [file pnas.2109123118.sapp.pdf]

PNAS
www.pnas.org

Supplementary Information for

High-dimensional profiling reveals phenotypic heterogeneity and disease-specific alterations of granulocytes in COVID-19

Magda Lourda[1,2], Majda Dzidic[1], Laura Hertwig[1], Helena Bergsten[1], Laura M. Palma Medina[1], Indranil Sinha[2], Egle Kvedaraite[1,2], Puran Chen[1], Jagadeeswara R. Muvva[1], Jean-Baptiste Gorin[1], Martin Cornillet[1], Johanna Emgård[1], Kirsten Moll[1], Marina García[1], Kimia T. Maleki[1], Jonas Klingström[1], Jakob Michaëlsson[1], Malin Flodström-Tullberg[1], Susanna Brighenti[1], Marcus Buggert[1], Jenny Mjösberg[1], Karl-Johan Malmberg[1], Johan K. Sandberg[1], Jan-Inge Henter[2,3], Elin Folkesson[4,5], Sara Gredmark-Russ[1,4], Anders Sönnerborg[4,6], Lars I. Eriksson[7,8], Olav Rooyackers[8,9], Soo Aleman[4,6], Kristoffer Strålin[4,6], Hans-Gustaf Ljunggren[1], Niklas K. Björkström[1], Mattias Svensson[1], Andrea Ponzetta[1], Anna Norrby-Teglund[1], Benedict J. Chambers[1]; and the Karolinska KI/K COVID-19 Study Group

Magda Lourda
Email: magdalini.lourda@ki.se

This PDF file includes:

Figures S1 to S7

Tables S1-S7

Legends for Datasets S1 and S2

Extended Materials and Methods

Additional References

Karolinska KI/K COVID-19 consortium details

Other supplementary materials for this manuscript include the following:

Dataset S1 and S2 (separate files)

Supplemental Figures

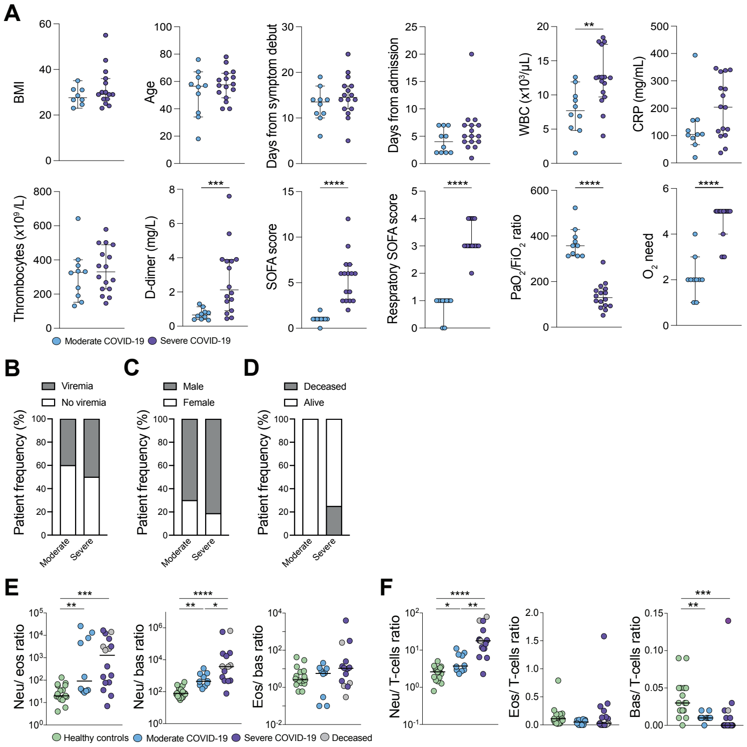

Figure S1. Granulocyte cell count ratios in COVID-19. (**A-D**) Clinical variables and demographic characteristics in patients with moderate (n=10) or severe COVID-19 (n=16). (**E**) Cell count ratios of granulocyte subsets (neu, neutrophils; eos, eosinophils; bas, basophils) and (**F**) of granulocyte subsets versus T-cells in healthy controls (n=17) and patients with moderate (n=10) or severe COVID-19 (n=16). Cell counts were based on Trucount flow cytometry analysis. Significant differences were evaluated with Kruskall-Wallis test and two-stage Benjamini, Krieger and Yekutieli test. FDR adjusted p-values are indicated**.** * $p < 0.05$; ** $p < 0.01$; *** $p < 0.001$; **** $p < 0.0001$.

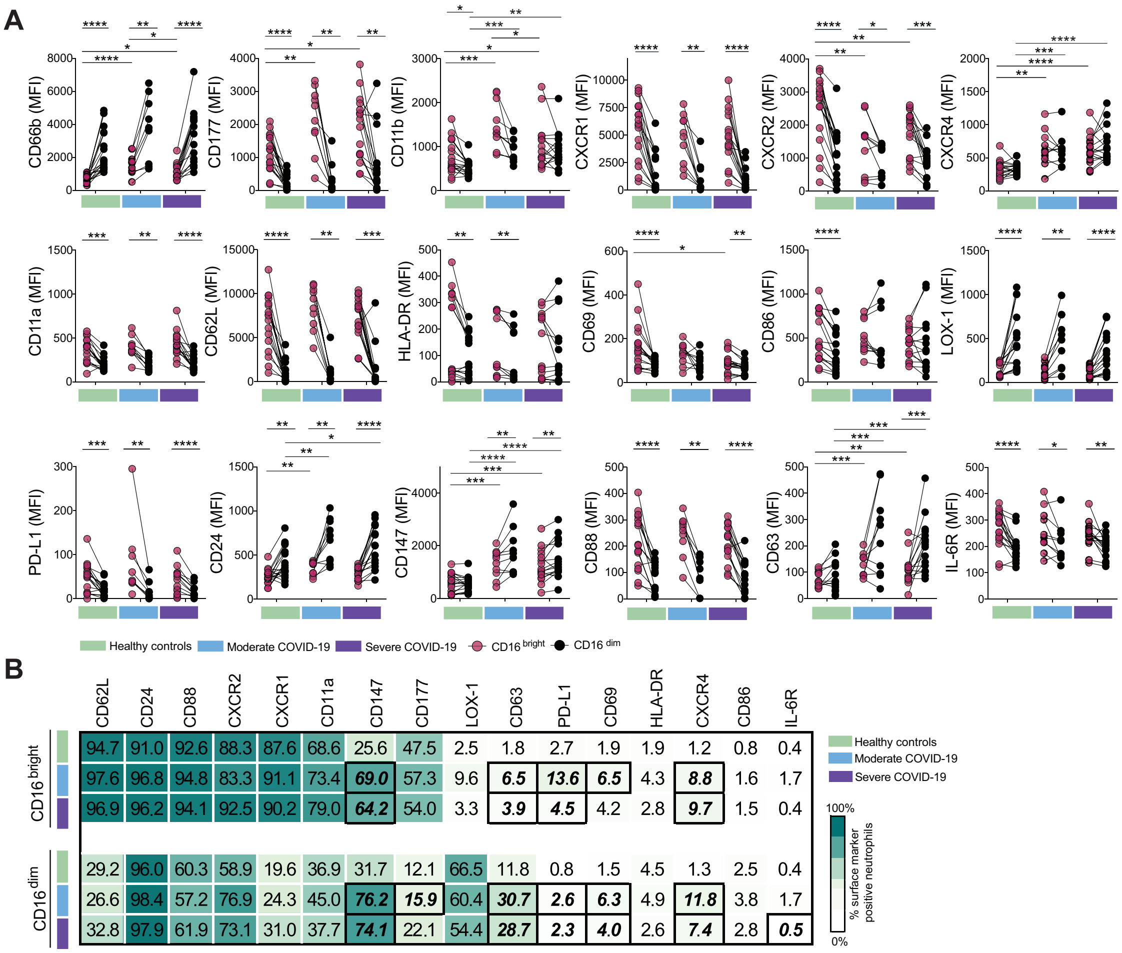

Figure S2. Neutrophil phenotypic characterization in COVID-19 patients. (**A**) Summary scatter plots showing the expression of the indicated markers on CD16^{bright} and CD16^{dim} neutrophils in healthy controls (n=17) moderate COVID-19 patients (n=10) and severe COVID-19 patients (n=16). (**B**) Heatmap showing the fraction within CD16^{bright} and CD16^{dim} neutrophils positive for the indicated markers in the three study groups. Mean values (%) are shown and indicated in bold italic if significantly different compared to healthy controls. In (**A**) Wilcoxon matched-pairs signed rank test was used to compare the expression in CD16^{bright} and CD16^{dim} subset within each patient and Kruskall-Wallis test with two-stage Benjamini, Krieger and Yekutieli test was used to compare marker expression across different experimental groups. In (**B**) Kruskall-Wallis test with two-stage Benjamini, Krieger and Yekutieli test was used. FDR adjusted p-values are indicated. * $p < 0.05$; ** $p < 0.01$; *** $p < 0.001$; **** $p < 0.0001$.

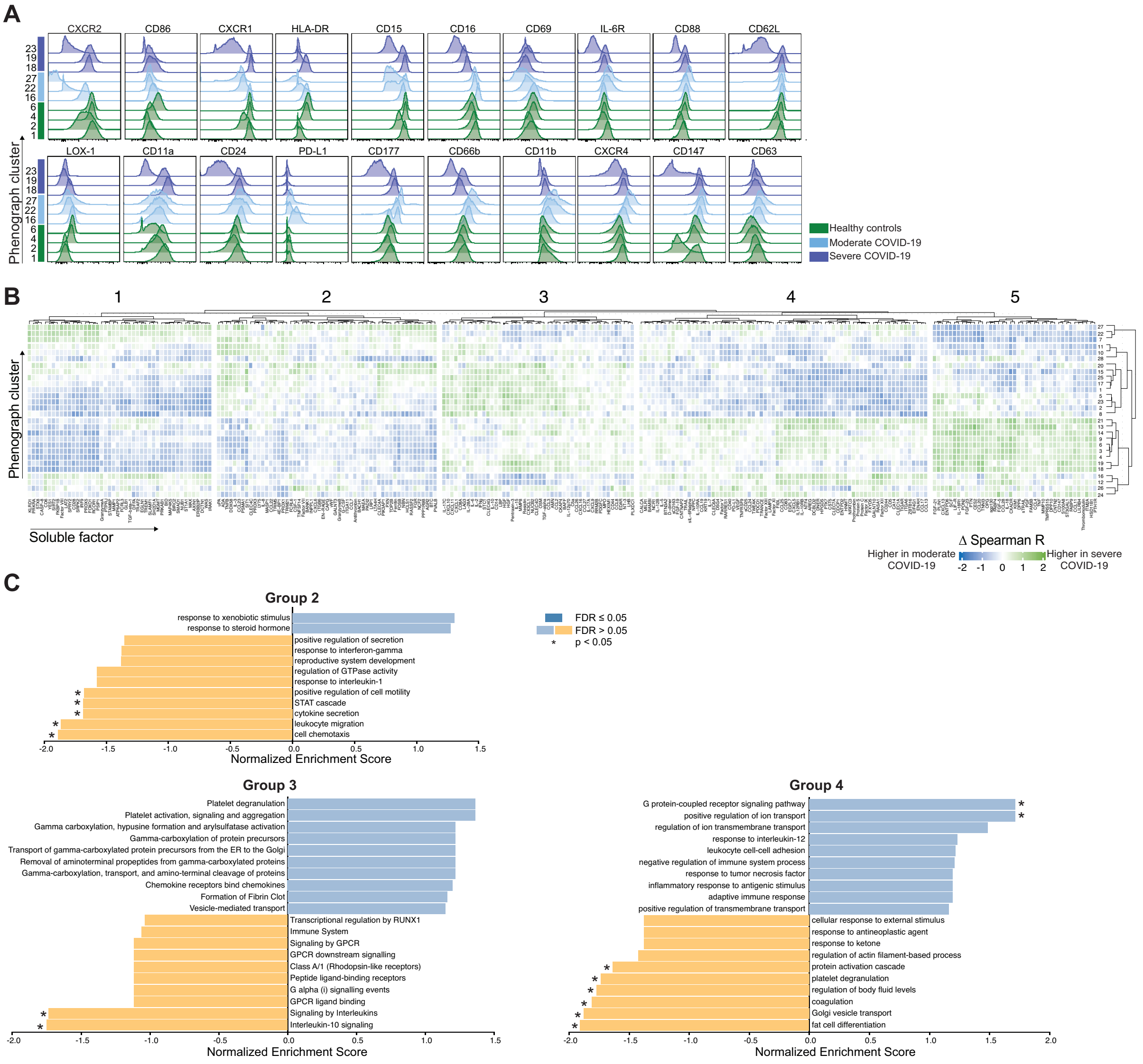

Figure S3. Phenograph analysis highlights phenotypic neutrophil heterogeneity and distinct association patterns with soluble factors in moderate and severe COVID-19 patients. (**A**) Representative histograms showing indicated marker expression for phenograph clusters shown in Figures 3A-B. (**B**) Heatmap showing the residuals of Spearman r correlation values between phenograph cluster frequencies in each individual and the serum/plasma level of the indicated soluble factors observed in severe minus that observed in moderate COVID-19 patients. The derived residual value (Δ Spearman R) ranges from -2 to +2, and indicates a higher correlation observed in either moderate (when closer to -2) or severe (when closer to +2) COVID-19 of the indicated cluster/soluble factor pairs. To highlight sets of specific associations between cluster frequencies and soluble factors in moderate versus severe COVID-19 patients, the matrix was split by *k*-means clustering. Raw Δ Spearman r values are shown in (B) and provided in detail in Table S5. (**C**) Pathway analysis (Reactome database) based on Spearman r values obtained from correlation between soluble factors and phenograph cluster frequencies, as displayed in Figure 3D (see Methods). Only pathways displaying a normalized enrichment score < -1 or > 1 are shown. * p < 0.05. Dark blue box indicates FDR < 0.05.

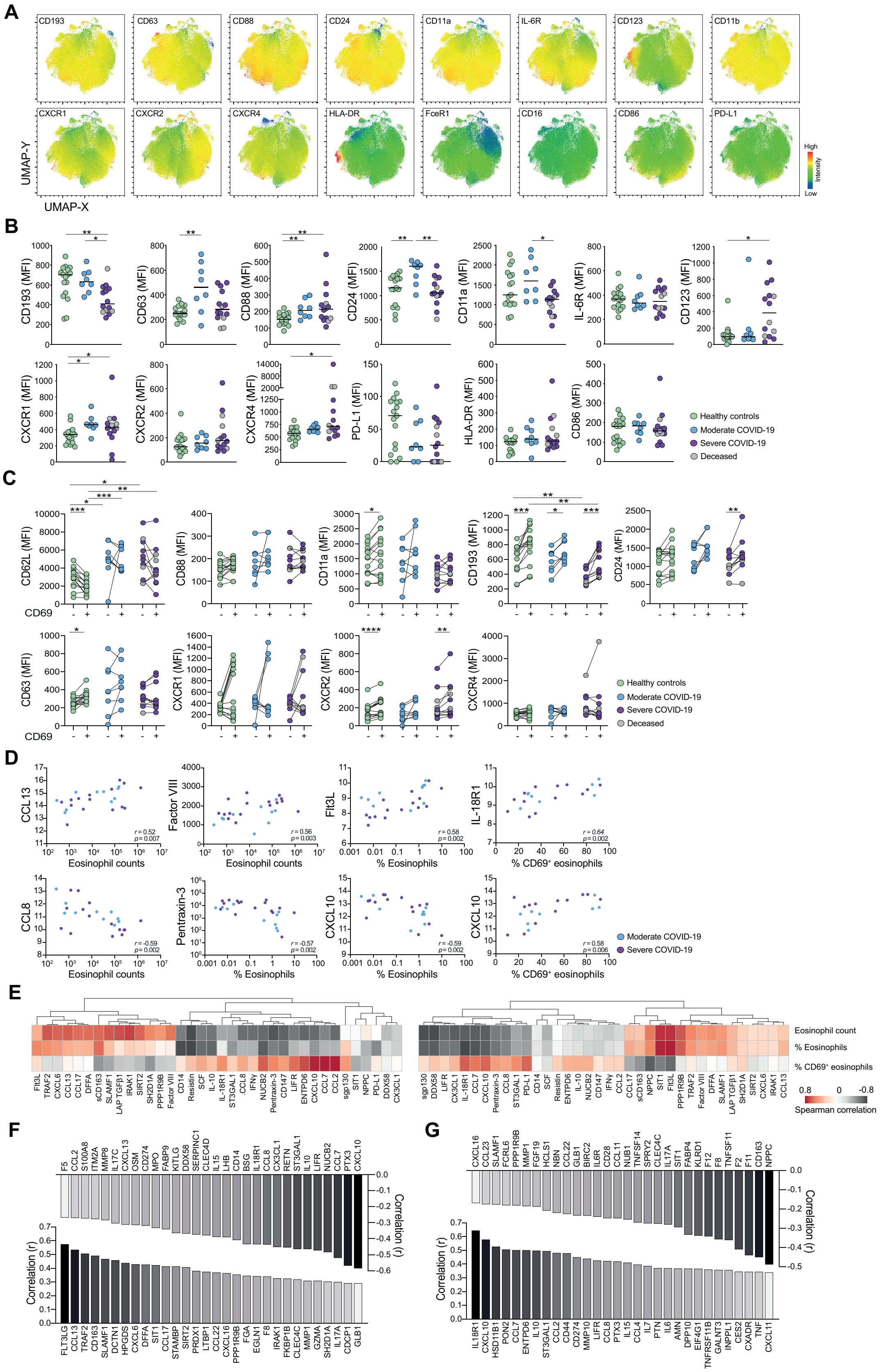

A
CD193
CD63
CD88
CD24
CD11a
IL-6R
CD123
CD11b
CXCR1
CXCR2
CXCR4
HLA-DR
FceR1
CD16
CD86
PD-L1
UMAP-Y
UMAP-X
High
Intensity
Low
B
CD193 (MFI)
CD63 (MFI)
CD88 (MFI)
CD24 (MFI)
CD11a (MFI)
IL-6R (MFI)
CD123 (MFI)
CXCR1 (MFI)
CXCR2 (MFI)
CXCR4 (MFI)
PD-L1 (MFI)
HLA-DR (MFI)
CD86 (MFI)
Healthy controls
Moderate COVID-19
Severe COVID-19
Deceased
C
CD62L (MFI)
CD88 (MFI)
CD11a (MFI)
CD193 (MFI)
CD24 (MFI)
CD69
CD63 (MFI)
CXCR1 (MFI)
CXCR2 (MFI)
CXCR4 (MFI)
Healthy controls
Moderate COVID-19
Severe COVID-19
Deceased
D
CCL13
Factor VIII
Flt3L
IL-18R1
r = 0.52 p = 0.007
r = 0.56 p = 0.003
r = 0.58 p = 0.002
r = 0.64 p = 0.002
Eosinophil counts
% Eosinophils
% CD69⁺ eosinophils
CCL8
Pentraxin-3
CXCL10
CXCL10
r = -0.59 p = 0.002
r = -0.57 p = 0.002
r = -0.59 p = 0.002
r = 0.58 p = 0.006
Moderate COVID-19
Severe COVID-19
E
Eosinophil count
% Eosinophils
% CD69⁺ eosinophils
Flt3L TRAF2 CXCL6 CCL13 CCL17 DFFA sCD163 SLAMF1 LAP TGFβ1 IRAK1 SIRT2 SH2D1A PPP1R9B Factor VIII CD14 Resistin SCF IL-10 IL-18R1 ST3GAL1 CCL8 IFNγ NUCB2 Pentraxin-3 CD147 LIFR ENTPD6 CXCL10 CCL7 CCL2 sgp130 SIT1 NPPC PD-L1 DDX58 CX3CL1
sgp130 DDX58 LIFR CX3CL1 IL-18R1 CCL7 CXCL10 Pentraxin-3 CCL8 ST3GAL1 PD-L1 CD14 SCF Resistin ENTPD6 IL-10 NUCB2 CD147 IFNγ CCL2 CCL17 sCD163 NPPC SIT1 Flt3L PPP1R9B TRAF2 Factor VIII DFFA SLAMF1 LAP TGFβ1 SH2D1A SIRT2 CXCL6 IRAK1 CCL13
0.8 0 -0.8
Spearman correlation
F
F5 CCL2 S100A8 ITM2A MMP8 IL17C CXCL13 OSM CD274 MPO FABP9 KITLG DDX58 SERPINC1 CLEC4D IL15 LHB CD14 BSG IL18R1 CCL8 CX3CL1 RETN ST3GAL1 IL10 LIFR NUCB2 CCL7 PTX3 CXCL10
Correlation (r)
FLT3LG CCL13 TRAF2 CD163 SLAMF1 DCTN1 HPGDS CXCL6 DFFA SIT1 CCL17 STAMBP SIRT2 PRDX1 LTBP1 CCL22 CXCL16 PPP1R9B FGA EGLN1 F8 IRAK1 FKBP1B CLEC4C MMP1 GZMA SH2D1A IL17A CDCP1 GLB1
Correlation (r)
G
CXCL16 CCL23 SLAMF1 FCRL6 PPP1R9B MMP1 FGF19 HCLS1 NBN CCL22 GLB1 BIRC2 IL6R CD28 CCL11 NUB1 TNFSF14 SPRY2 CLEC4C IL17A SIT1 FABP4 KLRD1 F12 F8 TNFSF11 F2 F11 CD163 NPPC
Correlation (r)
IL18R1 CXCL10 HSD11B1 PON2 CCL7 ENTPD6 IL10 ST3GAL1 CCL2 CD44 CD274 MMP10 LIFR CCL8 PTX3 IL15 CCL4 IL7 PTN IL6 AMN DPP10 EIF4G1 TNFRSF11B GALNT3 INPPL1 CES2 CXADR TNF CXCL11
Correlation (r)

Figure S4. Eosinophil phenotype and correlation with soluble factors in moderate and severe COVID-19 patients. (**A**) Overall UMAP displaying the MFI of the indicated receptors on eosinophils. (**B**) Scatter plots showing the MFI of eosinophil receptors in healthy controls (n=17) and in patients with moderate (n=8) and severe (n=14) COVID-19. The median value for each group is indicated. The deceased patients are indicated in gray. (**C**) Scatter plots showing the MFI of selected receptors in $CD69^-$ and $CD69^+$ eosinophil subsets in moderate (n=8) and severe (n=11) COVID-19 patients compared to healthy controls (n=15). (**D**) Correlation of eosinophil absolute counts or frequencies with indicated soluble factors. (**E**) Heatmaps showing significant correlations ($r < -0.4$ or $r > 0.4$, $p < 0.05$) between absolute eosinophil counts/percent of eosinophils in total leukocytes/percent of $CD69^+$ eosinophils and selected soluble factors in patients with moderate or severe COVID-19. (F-G) Bar graphs illustrating the Spearman r values of the top 30 positively correlated and the top 30 negatively correlated soluble factors with the frequency of total eosinophils (**F**) or the frequency of the $CD69^+$ eosinophils (**G**) that were used for the pathway analysis in Figure 4J. Significant differences between healthy controls and patient groups in (**B**) and (**C**) were evaluated with Kruskall-Wallis test and two-stage Benjamini, Krieger and Yekutieli test. (**C**) Significant differences between paired $CD69^-$ and $CD69^+$ subsets in the same sample were evaluated with Wilcoxon matched-pairs signed rank test. Significant correlations in (**D-E**) were evaluated with Spearman non-parametric test. FDR adjusted p-values are indicated. * $p < 0.05$; ** $p < 0.01$; *** $p < 0.001$.

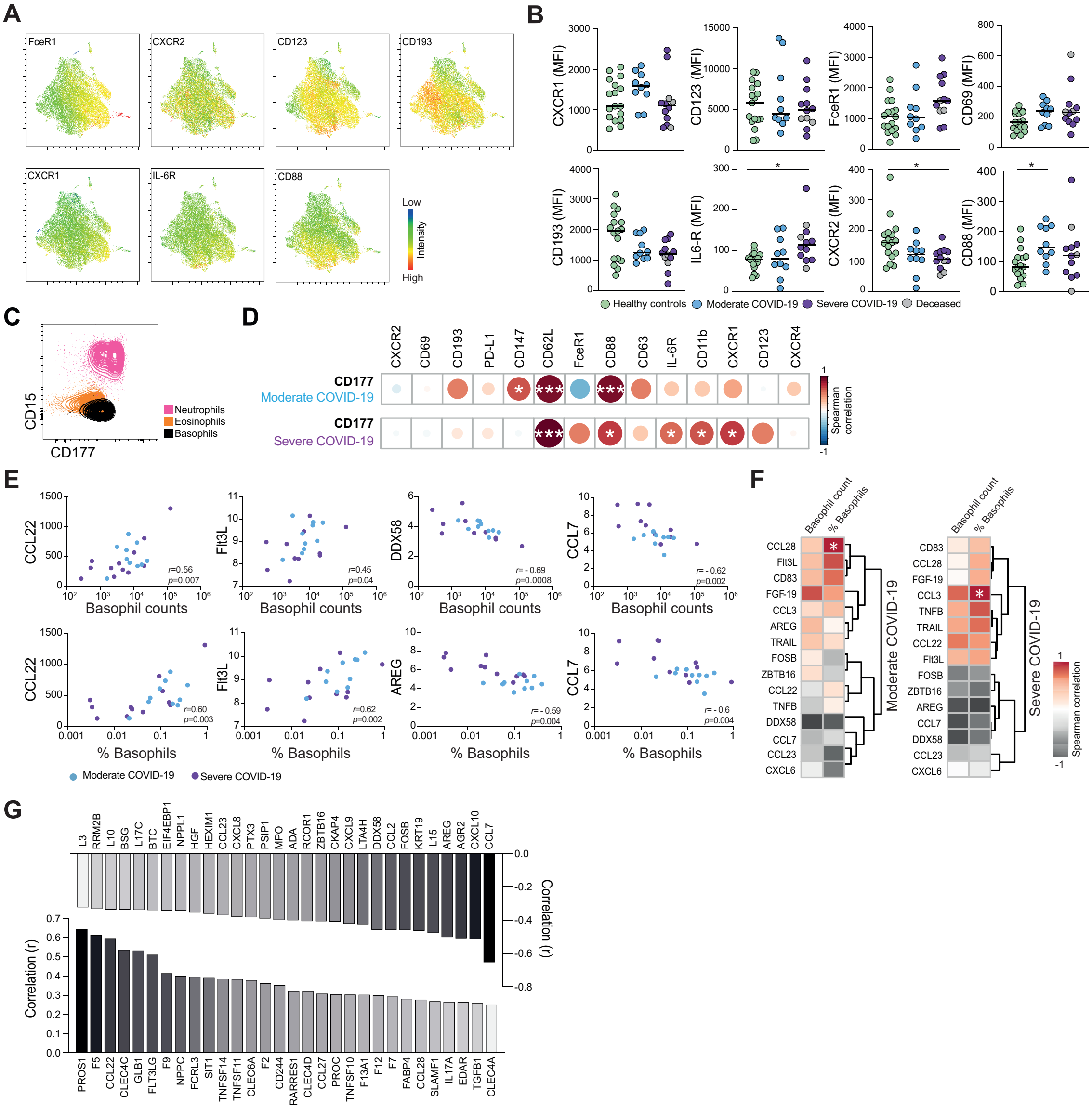

Figure S5. **Basophil phenotype and relation to circulating soluble factors in COVID-19 patients**. (**A**) Basophil UMAP showing the expression intensity of the indicated markers. (**B**) MFI for selected markers in basophil cell population in heathy controls (n=17), moderate (n=10) and severe COVID-19 patients (n=12). Bars show median values. (**C**) Representative dot plot of CD177 expression in basophils, eosinophils and CD16^{bright} neutrophils. (**D**) Correlation between expression of CD177 and indicated surface markers in basophils from COVID-19 patients. (**E**) Correlations between basophil absolute counts/frequencies and soluble factor concentrations in moderate and severe COVID-19. (**F**) Heatmaps showing correlation between absolute basophil counts/frequencies and soluble factor concentrations, in moderate and severe COVID-19. (G) Bar graph illustrating the Spearman r values of the top 30 positively correlated and the top 30 negatively correlated soluble factors with the absolute basophil counts that were used for the pathway analysis in Figure 5F. Significant differences between healthy controls and patient groups in (**B**) were evaluated with Kruskall-Wallis test and two-stage Benjamini, Krieger and Yekutieli test. Significant correlations in (**D-F**) were evaluated with Spearman non-parametric test. p-values are indicated**.** * $p < 0.05$, ** $p < 0.01$; *** $p < 0.001$.

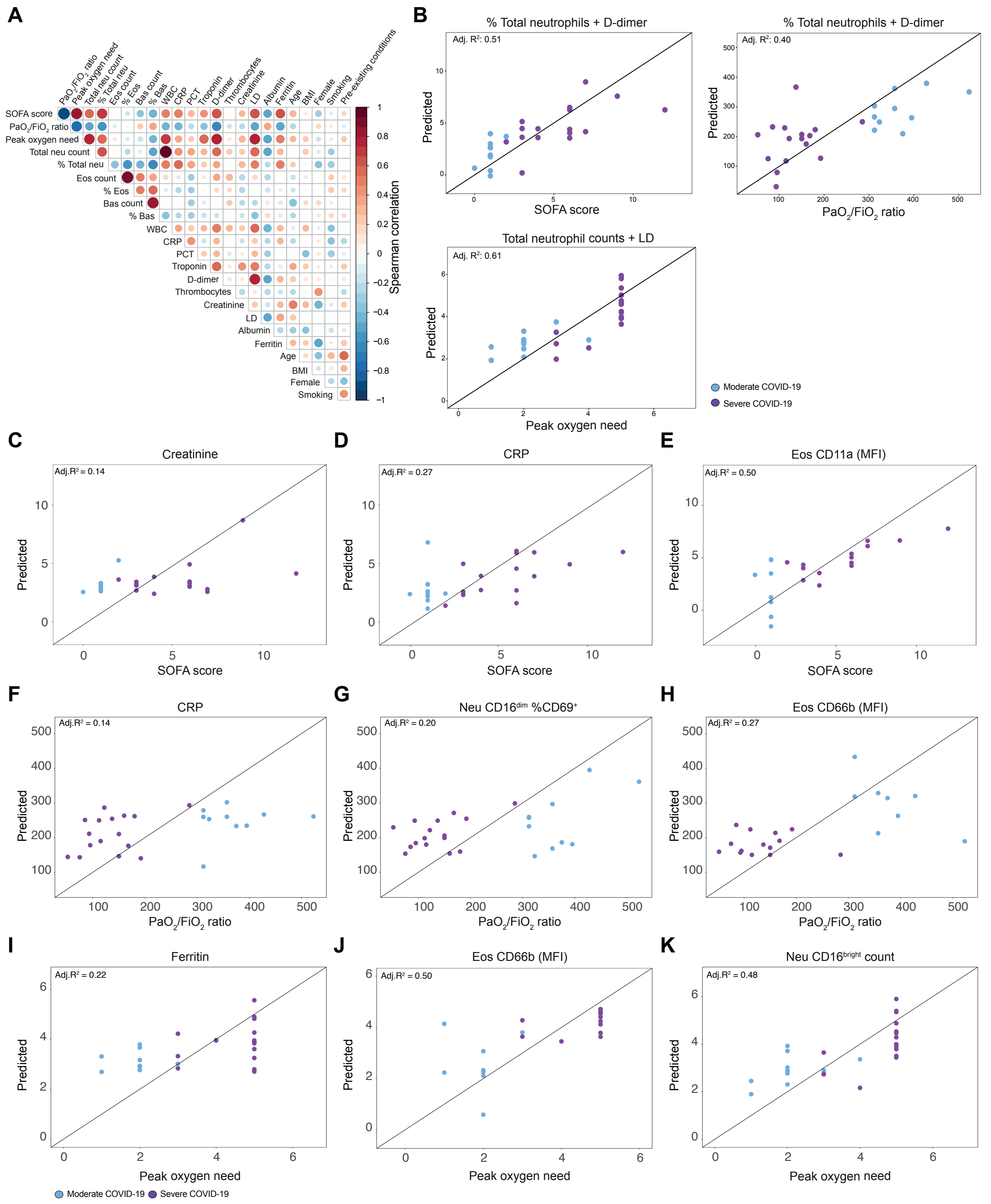

Figure S6. Neither immunologic nor clinical measurements alone are sufficient to predict relevant clinical outcomes. (**A**) Diagonal correlation matrix including predicted clinical outcomes and available clinical parameters. (**B**) Scatter plots of actual values versus predicted values of the best linear models constructed based only on available clinical parameters. Root mean square error and p-values of the models are available in Table S7. (**C-K**) Scatter plots of actual values versus predicted values of the linear models for prediction of SOFA score (**C-E**), PaO2/FiO2 ratio (**F-H**) and peak oxygen need (**I-K**) were modeled based on individual variables. Adj R^2, Adjusted R^2; Eos, Eosinophils; Neu, Neutrophils; Bas: Basophils; WBC: white blood cells; CRP: C-reactive protein; PCT: Procalcitonin; LD: lactate dehydrogenase; BMI: body mass index.

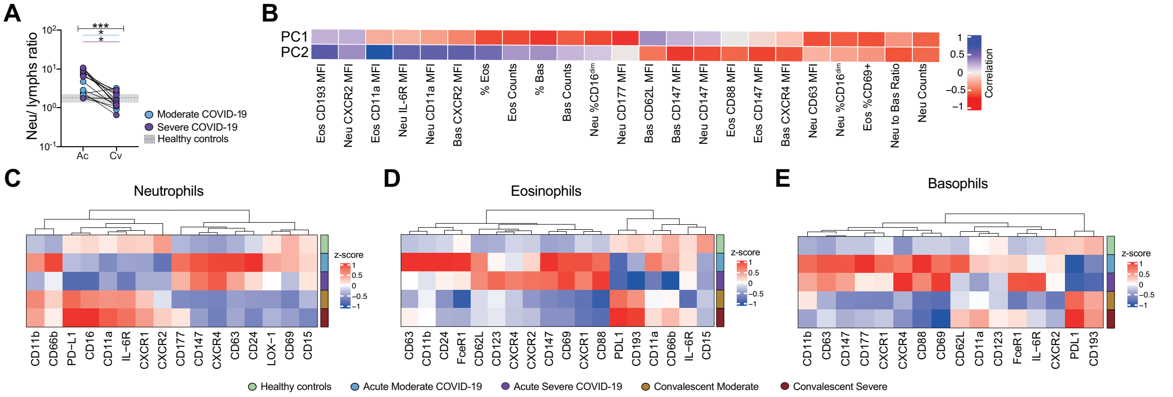

Figure S7. Granulocyte phenotype in paired acute and convalescent samples from COVID-19 patients. (**A**) Neutrophil (Neu) to lymphocyte (lymphs) ratio, based on absolute cell counts (as determined by Trucount flow cytometry approach), in paired acute (Ac) and convalescent (Cv) patient samples (n=15). Healthy controls (n=28, median±IQR) ranges are shown in gray. Bars indicate statistical significance considering all sampled patients (black), only moderate (blue) or only severe (purple) patients. (**B**) Contribution of individual variables, based on correlation coefficient, to PC1 and PC2 for PCA shown in Figure 7D-E. (**C**-**E**) Heatmaps showing average *z*-score values of all markers in healthy, acute moderate/severe and convalescent moderate/severe COVID-19 patients, for neutrophils (**C**), eosinophils (**D**) and basophils (**E**) respectively. In (**A**), Wilcoxon matched-pairs rank test; * $p < 0.05$; ** $p < 0.01$; *** $p < 0.001$; **** $p < 0.0001$.

Supplemental Tables

Table S1. Inclusion and exclusion criteria of COVID-19 patients and healthy controls.

		**Healthy controls n=17**	**Moderate COVID-19 n=10**	**Severe COVID-19 n=16**
**INCLUSION**	**Age**	18-78	18-78	18-78
	**Male**	12 (71%)	7 (70%)	13 (81%)
	**SARS-CoV-2 PCR**	Not assessed	Positive, from nasopharynx or sputum	Positive, from nasopharynx or sputum
	**Disease status**	Subjective health, no ongoing symptoms	Ongoing **hospital care** due to COVID-19 disease	Ongoing **intensive care** due to COVID-19 disease
	**Days from symptom onset to admission**		5-24 days	5-24 days
	**Days from hospital admission to sampling**		0-8 days	0-8 days *
	**Respiratory function**	Not assessed	Blood oxygen saturation 90-94% at screening. If supplemental oxygen flow, maximum: 3 L/min	Ongoing mechanical ventilation
	**Pre-existing health conditions**	Not assessed	No ongoing malignancy	No ongoing malignancy
	**Ongoing medications**	Not assessed	Low level corticosteroids and new drugs aimed specifically at COVID-19 were allowed, but no other immunosuppressant drugs.	Low level corticosteroids and new drugs aimed specifically at COVID-19 were allowed, but no other immunosuppressant drugs.
**EXCLUSION**	**SARS-CoV-2 serology**	Positive		

* one case was sampled at later time point, 20 days after admission

Table S2. Clinical characteristics of COVID-19 patients[1].

Clinical parameters	Moderate COVID-19 n=10	Severe COVID-19 n=16 #	p-value*
**Risk factors**			
**Age**	56 (18-76)	58 (40-78)	0.49
**Male (sex)**	7 (70%)	13 (81%)	0.64
**Smoking**	3 (60%) (n=5)	7 (47%) (n=15)	>0.99
**Body mass index**	27.6 (23-35)	29 (23-55)	0.33
**Co-morbidity**			
**None**	4 (40%)	5 (31%)	0.69
**Type 2 diabetes mellitus**	3 (30%)	4 (25%)	>0.99
**Hypertensive heart disease**	2 (20%)	4 (25%)	>0.99
**Coronary heart disease**	2 (20%)	2 (13%)	0.63
**Asthma**	1 (10%)	2 (13%)	>0.99
**Obesity**	0	2 (13%)	0.51
**Obstructive sleep apnea**	1 (10%)	1 (6%)	>0.99
**Chronic pain**	1 (10%)	1 (6%)	>0.99
**Days from symptom to admission**	8.5 (4-14)	9 (3-14)	0.95
**Days from symptom to sampling**	13.5 (6-17)	14.5 (5-24)	0.24
**Viremia at sampling**	4 (40%)	7 (44%)	>0.99
**Serology at sampling**			
**Positive (>=12 AU/mL)**	5 (50%)	13 (81%)	
**Borderline (8-11.99 AU/mL)**	2 (20%)	2 (20%)	
**Negative (<8 AU/mL)**	3 (30%)	1 (6%)	
**SARS-CoV-2 IgG (AU/mL)**	19 (0.26-106)	55 (3.4-211)	0.17
**Symptoms at admission**			
**Fever**	10 (100%)	16 (100%)	>0.99
**Cough**	9 (90%)	13 (81%)	>0.99
**Dyspnea**	10 (100%)	16 (100%)	>0.99
**Myalgia**	3 (30%)	8 (50%)	0.43

**Gastrointestinal symptoms**	1 (10%)	3 (19%)	>0.99
**Embolism/thrombosis**	0 (0%)	3 (19%)	0.27
**Positive blood culture**	0 (0%)	3 (19%)†	0.27
**Positive lower respiratory tract culture**	0 (0%)	6 (38%)‡	0.05
**Peak supportive oxygen therapy**			
**None**	2 (20%)	0 (0%)	0.14
**Supportive oxygen**	8 (80%)	4 (25%)	0.01
**Ventilator**	0 (0%)	11 (69%)	0.0007
**ECMO**	0 (0%)	1 (6%)	>0.99
**PaO_2/FiO_2 (mmHg)**	357 (312-523)	130 (52-285)	<0.0001
**SOFA score**	1 (0-1)	6 (3-12)	<0.0001
**Treatment prior to sampling**			
**Anticoagulant prophylaxis**	Low mw heparin (n=10; 100%)	Low mw heparin (n=14), oral anticoagulants (n=1), total 93%	>0.99
**Antibiotics**	3 (30%): cefotaxime	12 (75%): cefotaxime, piperacillin/tazobactam	0.04
**Corticosteroids**	2 (20%)	12 (75%)	0.0001
**Off-label drugs**	0 (0%)	2 (13%): Tocilizumab, Anakinra	>0.99
**Outcome**			
**Alive**	10 (100%)	12 (75%)	0.14
**Days of supportive oxygen therapy**	2.5 (0-108)	20 (7-170)§	0.001

[1] Unless otherwise stated, all parameters are indicated as median value (range/%).

# One of the severe patients was treated in a high dependency unit at sampling with extracorporeal membrane oxygenation (ECMO). The rest of the severe patients (n=15) were treated in the Intensive care unit (ICU).

§ One patient was treated with ECMO for 170 days before being transferred to another hospital for lung transplantation.

* Statistical significance was determined by Mann-Whitney test or Fisher's exact test.

† *Staphylococcus aureus (n=1), Streptococcus milleri (n=1), S. aureus + Enterococcus faecalis (n=1)*

‡ *S. aureus* (n=2, *Candida albicans (n=1)*, *Escherichia coli (n=1)*, S. aureus + *Streptococcus dysgalactiae (n=1), S. aureus + C. albicans + Klebsiella pneumonia + Aspergillus fumigatus (n=1)*

Abbreviations:

ECMO, Extracorporeal membrane oxygenation; SOFA, Sequential organ failure assessment; mw, molecular weight.

Table S3. Laboratory characteristics of COVID-19 patients.

Laboratory parameters	Reference	Moderate COVID-19	Severe COVID-19	p-value*
C-reactive protein (mg/L)	<3	104 (20-394) (n=9)	204 (37-346)	0.14
Procalcitonin (μg/L)	<0.5	0.40 (0.12-886) (n=9)	0.61 (0.16-10)	0.40
Leukocytes (x10^9/L)	3.5-8.8	7.7 (1.5-12.6) (n=7)	12.5 (4-18.4)	0.007
Neutrophils (x10^9/L)	1.6-5.9	5.4 (0.9-9.3)	10.9 (3.2-16.7)	0.001
Lymphocytes (x10^9/L)	1.1-3.5	1.2 (0.3-2.4)	0.7 (0-3-2.3)	0.03
Thrombocytes (x10^9/L)	165-387	324 (131-642) (n=9)	331 (146-579)	0.58
D-dimer (mg/L)	<0.56	0.71 (0.36-1.3) (n=9)	2.1 (0.44-5.4)	0.0009
Fibrinogen (g/L)	2-4.2	n/a	6.3 (n=14) (3.5-10.2)	n/a
Troponin T (ng/L)	<15	6 (5-43) (n=7)	17.5 (6-434)	0.01
Creatinine (μmol/L)	<90	67.5 (43-168)	76 (36-326)	0.34
Lactate dehydrogenase (μkat/L)	<3.5	5.55 (3.8-13.7)	10.6 (0.89-23)	0.003
Albumin (g/L)	36-48	27 (21-37) (n=7)	19.5 (16-29)	0.05
Myoglobin (μg/L)	<73	23 (22-37) (n=3)	369.5 (36-6400) (n=15)	n/a
Interleukin-6 (ng/L)	<7	53 (2-151) (n=10)	174 (29-8093)	0.004
Interleukin-1β (ng/L)	<5	<5 (n=1)	<5 (<5-16.8) (n=15)	n/a
Interleukin-10 (ng/L)	<5	n/a	24 (8.8-47.7) (n=15)	n/a
Tumor necrosis factor-α (ng/L)	<12	n/a	15.5 (8-39.5) (n=15)	n/a

[1] Unless otherwise stated, all parameters are indicated as median value (range).

[2] Laboratory parameters were measured +/- 24h from sampling. Interleukins and tumor necrosis factor were measured +/- 5 days from sampling. If group contain missing data, number of patients for which data was obtained is indicated as ***n*** within cell.

*Statistical significance was determined by Mann-Whitney test.

Table S4. Efficacy of prediction models after correction for the indicated variables.

Clinical parameters		p-value*
**SOFA**	Female	0.316
	Age	0.062
	BMI	0.261
	Days from symptom to sampling	0.606
	Smoking	0.362
	Days from admission to sampling	0.298
	Co-morbidities	0.828
**PaO_2/FiO_2 ratio**	Female	0.700
	Age	0.275
	BMI	0.537
	Days from symptom to sampling	0.605
	Smoking	0.069
	Days from admission to sampling	0.805
	Co-morbidities	0.214
**Peak oxygen**	Female	0.925
	Age	0.850
	BMI	0.383
	Days from symptom to sampling	0.396
	Smoking	0.602
	Days from admission to sampling	0.240
	Co-morbidities	0.508

*Anova test.

Table S5. Summary of the results from the constructed linear models.

		Linear model		
		Adj. R^2	RMSE	p-value
**Models with clinical parameters and granulocyte traits**				
Linear model for SOFA	Eos CD11a (MFI) + CRP + Creatinine	0.74	1.45	3.88E-06
	Eos CD11a (MFI)	0.50	2.12	1.37E-04
	CRP	0.27	2.46	3.60E-03
	Creatinine	0.14	2.67	3.33E-02
Linear model for PaO_2/FiO_2 ratio	Eos CD66b (MFI) + Neu $CD16^{dim}$ $\%CD69^{+}$ cells + CRP	0.60	78.33	1.92E-04
	Eos CD66b (MFI)	0.27	111.48	7.62E-03
	Neu $CD16^{dim}$ $\%CD69^{+}$ cells	0.20	111.60	1.20E-02
	CRP	0.14	116.09	3.47E-02
Linear model for Peak Oxygen needed	Neu $CD16^{bright}$ count + Eos CD66b (MFI) + Ferritin	0.74	0.70	3.97E-06
	Neu $CD16^{bright}$ count	0.48	1.02	4.79E-05
	Eos CD66b (MFI)	0.50	1.03	1.49E-04
	Ferritin	0.22	1.25	8.71E-03
**Models with only clinical parameters**				
Linear model for SOFA	% Total neu + D-dimer	0.51	1.97	9.57E-05
	% Total neu	0.36	2.30	6.71E-04
	D-dimer	0.34	2.34	1.06E-03
Linear model for PaO_2/FiO_2 ratio	% Total neu + D-dimer	0.40	94.90	1.09E-03
	% Total neu	0.31	104.03	1.92E-03
	D-dimer	0.24	108.82	6.17E-03
Linear model for Peak Oxygen needed	Total neu counts + LD	0.61	0.87	8.53E-06
	Total neu counts	0.48	1.02	5.58E-05
	LD	0.45	1.05	1.13E-04

Abbreviations:

Adj R^2: Adjusted R^2; RMSE: Root mean square error; SOFA: sequential organ failure assessment; Eos: eosinophils; CRP: C-reactive protein; Neu: Neutrophils; LD: lactate dehydrogenase.

Table S6. Results of the receiver operating characteristic (ROC) analysis performed with severity as outcome.

	**ROC analysis**			
	AUC	[95% CI]	Sensitivity	Specificity
**Selected cellular populations - Individual**				
CRP	0.68	0.45-0.91	0.69	0.70
Creatinine	0.62	0.39-0.84	0.50	0.90
Ferritin	0.76	0.57-0.95	0.63	0.90
Eos CD11a (MFI)	0.82	0.62-1	0.86	0.75
Eos CD66b (MFI)	0.92	0.8-1	1.00	0.75
Neu CD16^{bright} count	0.81	0.63-0.98	0.81	0.80
Neu CD16^{dim} %CD69^{+} cells	0.63	0.37-0.88	0.69	0.60
**Lineal models with cellular populations**				
Eos CD11a (MFI) + CRP + Creatinine	0.95	0.86-1	0.86	1.00
Eos CD66b (MFI) + Neu CD16^{dim} %CD69^{+} cells + CRP	1.00	1-1	1.00	1.00
Neu CD16^{bright} count + Eos CD66b (MFI) + Ferritin	0.97	0.91-1	0.93	1.00
**Clinical parameters - Individual**				
Total neu count	0.82	0.65-0.98	0.81	0.80
% Total neu	0.79	0.61-0.97	0.63	0.80
Eos count	0.42	0.18-0.66	0.56	0.60
% Eos	0.52	0.26-0.77	0.81	0.50
Bas count	0.73	0.52-0.93	0.69	0.80
% Bas	0.81	0.64-0.98	0.69	0.90
WBC	0.79	0.61-0.96	0.69	0.90
PCT	0.61	0.35-0.86	0.81	0.50
Troponin	0.80	0.6-0.99	0.94	0.60
D-dimer	0.88	0.74-1	0.75	1.00
Thrombocytes	0.57	0.33-0.81	0.50	0.70
LD	0.84	0.67-1	0.81	0.90
Albumin	0.73	0.51-0.95	0.69	0.80
Age	0.58	0.34-0.83	0.44	0.70
BMI	0.63	0.38-0.88	0.67	0.63
Female	0.44	0.26-0.62	0.19	0.70
Smoking	0.57	0.29-0.84	0.53	0.60
**Lineal models with only clinical Parameters**				
% Total neu + D-dimer	0.93	0.8-1	0.88	1.00
Total neu count + LD	0.93	0.8-1	0.88	1.00
% Total neu + D-dimer	0.84	0.68-1	0.75	0.90

Abbreviations:

AUC: area under the curve; CI: confidence interval; CRP: C-reactive protein; neu: neutrophils; Eos: eosinophils; Neu: Neutrophils; Bas: basophils; WBC: white blood cells; PCT: Procalcitonin; LD: lactate dehydrogenase; BMI: body mass index.

Table S7. Flow cytometry panel used in the study.

Antigen	Clone	Fluorochrome	Laser line	Filter	Dilution	Company
**CD16**	3G8	BUV805	***UV (355nm, 100mW)***	810/40	25	BD
**CD86**	2331 (FUN-1)	BUV737		735/30	50	BD
**CD62L**	DREG-56	BUV661		670/25	50	BD
**CD147**	HIM6	BUV615		605/20	25	BD
**PD-L1**	MIH1	BUV563		580/20	20	BD
**CD24**	ML5	BUV496		515/30	25	BD
**CD193**	5E8	BUV395		379/28	50	BD
**CD63**	H5C6	BV786	***Violet (405nm, 100mW)***	810/40	25	BD
**CD15**	W6D3	BV750		750/30	20	BD
**CD88**	D53-1473	BV711		710/50	25	BD
**CD11a**	HI111	BV650		677/20	50	BD
**FceR1**	AER-37	BV605		605/40	50	BD
**Dead Cell Stain**	N/A	Fixable yellow		586/15	400	ThermoFisher
**CD3**	SK7	BV510		525/50	50	BioLegend
**CD14**	M5E2				100	BioLegend
**CD19**	SJ25C1				50	BD
**CD56**	NCAM16.2				100	BD
**CD304**	U21-1283				50	BD
**LOX-1**	15C4	BV421		450/50	25	BioLegend
**CD69**	FN50	BB700	***Blue (488nm, 200nW)***	710/50	25	BD
**CD66b**	G10F5	BB515		530/30	200	BD
**CD123**	7G3	PE-Cy7	***Yellow/green (561nm, 200mW)***	780/60	20	BD
**CXCR4**	2B11	PE-Cy5.5		710/50	25	BD
**CXCR1**	8F1/CXCR1	PE-Cy5		670/30	20	BD
**CD11b**	ICRF44	PE-CF594		610/20	100	BD
**CD126**	M5	PE		586/15	50	BD
**CXCR2**	5E8/CXCR2	APC-Fire	***Red (637nm, 140mW)***	780/60	50	BioLegend
**HLA-DR**	G46-6	APC-R700		730/45	100	BioLegend
**CD177**	MEM-166	Alexa647		670/30	100	BioLegend

Dataset S1 (separate file). List of analytes measured by Olink or multiplex in serum or plasma. Analyte quantification results with more than 25% of out of range values were not suitable for imputation and are indicated with a star.

Dataset S2 (separate file). Raw delta Spearman correlation values between phenograph cluster frequencies and the concentration level of the indicated soluble factors.

Extended Materials and Methods

Proximity extension assay

The proximity extension assay technology (PEA), based on Q-PCR quantification of pair-wise binding of oligonucleotide-labeled target antibodies (OLINK AB, Uppsala, Sweden), was used for the quantification of 276 selected soluble factors in serum from the study participants (**Dataset S1**).

Luminex assays

Soluble factors in the serum of the study participants were measured using 4 customized multiplex panels including 36, 12, 15 and 12 analytes covering a range of human inflammatory factors (R&D Systems, UK; Dataset S1). Coagulation factors were measured in plasma using 3 different multiplex panels, including the 6-, 4-, and 3-plex human ProcartaPlex panels (ThermoFisher). Assays were performed according to the manufacturer's guidelines and samples were acquired on a Luminex MAGPIX instrument using xPonent 4.0 software (Luminex).

Flow cytometry data analysis

Standard flow cytometry data analysis was performed using FlowJo version 10. A compensation matrix for the 25-color flow cytometry panel was generated using AutoSpill (63), optimized and applied to all fcs files. All data was pre-processed using the time gate. Following exclusion of doublets and dead cells, neutrophils were defined as $CD15^{+}CD193^{-}CD66b^{+}CD16^{bright/dim}$, eosinophils were defined as $CD15^{bright/dim}CD193^{bright/dim}CD16^{-}FcER1^{dim}CD123^{dim/-}$ and basophils were defined as $CD15^{-}CD193^{bright}FcER1^{bright}CD123^{bright}HLA\text{-}DR^{dim/-}$. In addition, exclusion of potential contamination by non-granulocytes was done using lineage exclusion markers (CD3, CD14, CD19, CD56, CD304). Samples containing less than 30 events in the final granulocyte subset gate were not considered for further phenotypical analysis. The Cytonorm FlowJo plugin v1.0 was applied on each granulocyte population to correct for potential batch effects and was based on an internal control that was sampled on all sampling occasions. Dimensionality reduction was performed with the UMAP FlowJo plugin v3.1. Neutrophil populations from each individual were down-sampled for comparability (FlowJo Downsample plugin v3.3), barcoded and concatenated. FlowJo Phenograph v3 was used for unsupervised

clustering, with the optimal k-nearest neighbors (KNN) implemented automatically. Trying other KNN values did not affect the clustering of markers or the generated number of phenograph clusters.

Pathway analysis

Pathway analysis was based on the correlation between soluble factor serum concentration in each patient and frequency of phenograph clusters (for neutrophils) or frequencies or absolute counts for eosinophils and basophils respectively. The analysis was performed using the publicly available tool WebGestalt (http://www.webgestalt.org), from the original publication by Liao et al. (64). For neutrophils, the median Spearman r value for each cluster group (as indicated in Figure 3D) was calculated for individual cluster/soluble factor pair. For all granulocyte subsets, the values corresponding to the top 30 positively correlated and top 30 negatively correlated molecules were used to perform the Gene Set Enrichment Analysis (GSEA) according to the following parameters: Functional database = "pathway", "Reactome"; minimum number of genes for a category = 3; Significance level = Top 10; Number of permutations = 1000. Other parameters were used as in the default settings. Only pathways with at least >1 or <-1 enrichment score are shown in the corresponding figures.

Receptor-ligand interaction analysis

Publicly available scRNAseq data set, based on PBMC and whole blood cells (19), was utilized to analyze interactions between neutrophils and other circulating immune cells. *Seurat .RDS* file was acquired from FASTGENOMICS web site (https://beta.fastgenomics.org/home) and the dataset was divided into 3 sample groups, i.e., control, mild and severe, as reported in (19). Count matrices for each sample group were generated and ran through CellphoneDB pipeline (37). In Figure 3F, the population pairs were selected according to the total number of significant ligand-receptor interactions within the investigated ligand-receptor axes.

Multivariable linear regression

Predictive models for clinical outcomes were built based on a data set of 167 immune traits that included surface marker expression levels, absolute numbers, frequencies of granulocyte subsets, available clinical information and laboratory measured parameters. The creation of the models

was performed in R v. 3.6.0 (65). Variable redundancy was avoided by identification of variables with pair-wise Spearman correlation coefficient higher than 0.99 (function *cor* from package stats), and removal of the variable with the largest mean absolute correlation. The remaining variables were pair-wise correlated with the clinical outcome, and those with p-values < 0.05 were selected for further evaluation. All possible linear combinations of variables were evaluated with the function *regsubsets* from the package leaps (66) using an 'exhaustive' method and evaluating 3 subsets per model size. The models with highest adjusted R^2, lowest bayesian information criterion and a Mallow's Cp lower and closer to the number of coefficients, were selected for further evaluation. The variance inflation factor for each model was calculated to avoid redundancy in the independent variables with the function *vif* from package *car* (67). The selected final model had no redundant variables, and all must be significant (p-value < 0.05). The contribution of each parameter to each model was calculated with the functions *cal.relimp* from package *relaimpo* (68) with the lgm metric. Differences in the model after addition of factors for correction (sex, age, BMI, smoking, days from symptom to sampling and from admission to sampling and co-morbidities) were evaluated by computing an analysis of variance for the two linear models.

Receiver operator characteristic analysis

The predictive ability of the selected cellular population, clinical parameters and the resulting multivariable linear models was tested with receiver operating characteristic analysis having severity as dichotomous outcome. The tests were performed using the R package pROC (69) and the reported coordinated were selected as the point closest to the top-left part of the plot. The analysis of the multivariable models was performed by first creating a generalized linear model with binomial family (function *glm* from package *stats*).

Statistical analysis

GraphPad Prism version 9 (GraphPad Software) and R v4.0.1 (65) were used to conduct statistical analyses, where p-values < 0.05 were considered significant. Two-tailed and non-parametric Mann-Whitney *U* test was used for two-group comparisons. Non-parametric Kruskal-Wallis test, in combination with two-stage step-up method of Benjamini, Krieger and Yekutieli for controlling False Discovery rate (FDR), was used for multiple-group comparisons. Paired

samples were analyzed with Wilcoxon matched-pairs signed rank test. For correlations, the two-tailed non-parametric Spearman test was applied. Where indicated, -score of median fluorescence intensity (MFI) was calculated as follows: $z = (x-\mu)/\sigma$, being x = raw score, μ = mean of sample distribution and σ = standard deviation. Correlation, hierarchical clustering and multivariate analysis of flow cytometry data with clinical parameters and proteomic data was performed and visualized in GraphPad Software and R v4.0.1 (65), using the packages factoextra (v1.0.7) (70), FactoMineR (v2.3) (71), PerfomanceAnalytics (v2.0.4) (72), ggplot2 (v3.3.1) (73), gplots (v3.0.4) (74), pheatmap (v1.0.12) (75), vegan (v2.5-6) (76), corrplot (v0.84) (77), lattice (v0.20-41) (78) and latticeExtra (v0.6-29) (79), stats (v4.0.1) and complexheatmap (v2.5.6) (80).

Additional References

63. C. P. Roca *et al.*, AutoSpill: a method for calculating spillover coefficients in high-parameter flow cytometry. *bioRxiv* 10.1101/2020.06.29.177196, 2020.2006.2029.177196 (2020).
64. Y. Liao, J. Wang, E. J. Jaehnig, Z. Shi, B. Zhang, WebGestalt 2019: gene set analysis toolkit with revamped UIs and APIs. *Nucleic Acids Res* **47**, W199-W205 (2019).
65. R. C. Team, R: A language and environment for statistical computing. (2019).
66. T. Lumley, leaps: Regression Subset Selection. Based on Fortran code by Alan Miller. (2020).
67. J. Fox *et al.*, car: Companion to Applied Regression. (2020).
68. U. Groemping, L. Matthias, relaimpo: Relative Importance of Regressors in Linear Models. (2018).
69. X. Robin *et al.*, pROC: an open-source package for R and S+ to analyze and compare ROC curves. *BMC Bioinformatics* **12**, 77 (2011).
70. A. Kassambara, F. Mundt, factoextra: Extract and Visualize the Results of Multivariate Data Analyses. (2020).
71. S. Lê, J. Josse, F. Husson, FactoMineR: An R Package for Multivariate Analysis. *Journal of Statistical Software* **25**, 1-18 (2008).
72. B. G. Peterson *et al.*, PerformanceAnalytics: Econometric Tools for Performance and Risk Analysis. (2020).
73. H. Wickham, *ggplot2: Elegant Graphics for Data Analysis*, Use R! (Springer International Publishing, ed. 2, 2016).
74. G. R. Warnes *et al.*, gplots: Various R Programming Tools for Plotting Data. (2020).
75. R. Kolde, pheatmap: Pretty Heatmaps. (2019).
76. J. Oksanen *et al.*, vegan: Community Ecology Package. (2020).
77. T. Wei *et al.*, corrplot: Visualization of a Correlation Matrix. (2017).
78. D. Sarkar, Lattice: Multivariate Data Visualization with R. *Use R!* (2008).
79. D. Sarkar, F. Andrews, latticeExtra: Extra Graphical Utilities Based on Lattice. (2019).
80. Z. Gu, R. Eils, M. Schlesner, Complex heatmaps reveal patterns and correlations in multidimensional genomic data. *Bioinformatics* **32**, 2847-2849 (2016).

Karolinska KI/K COVID-19 consortium details

Mira Akber[1], Soo Aleman[2], Lena Berglin[1], Helena Bergsten[1], Niklas K Björkström[1], Susanna Brighenti[1], Demi Brownlie[1], Marcus Buggert[1], Marta Butrym[1], Benedict J Chambers[1], Puran Chen[1], Martin Cornillet[1], Angelica Cuapio[1], Isabel Diaz Lozano[1], Lena Dillner[2], Majda Dzidic[1], Johanna Emgård[1], Lars I Eriksson[3], Anna Färnert[2], Malin Flodström-Tullberg[1], Hedvig Glans[2], Jean-Baptiste Gorin[1], Sara Gredmark-Russ[1], Jonathan Grip[3], Alvaro Haroun-Izquierdo[1], Elisabeth Henriksson[1], Laura Hertwig[1], Sadaf Kalsum[1], Tobias Kammann[1], Jonas Klingström[1], Efthymia Kokkinou[1], Egle Kvedaraite[1], Hans-Gustaf Ljunggren[1], Marco Giulio Loreti[1], Magdalini Lourda[1], Kimia T Maleki[1], Karl-Johan Malmberg[1], Nicole Marquardt[1], Johan Mårtensson[3], Christopher Maucourant[1], Jakob Michaëlsson[1], Jenny Mjösberg[1], Kirsten Moll[1], Jagadeeswara Rao Muvva[1], Pontus Nauclér[2], Anna Norrby-Teglund[1], Laura M Palma Medina[1], Tiphaine Parrot[1], André Perez-Potti[1], Björn P Persson[3], Lena Radler[1], Emma Ringqvist[1], Olga Rivera-Ballesteros[1], Olav Rooyackers[3], Johan K Sandberg[1], John Tyler Sandberg[1], Takuya Sekine[1], Ebba Sohlberg[1], Tea Soini[1], Anders Sönnerborg[2], Kristoffer Strålin[2], Mattias Svensson[1], Janne Tynell[1], Christian Unge[4], Renata Varnaite[1], Andreas von Kries[1], David Wullimann[1]

[1]Center for Infectious Medicine, Department of Medicine Huddinge, Karolinska Institutet, Karolinska University Hospital, Stockholm, Sweden

[2]Department of Infectious Diseases, Karolinska University Hospital, Stockholm, Sweden

[3]Function Perioperative Medicine and Intensive Care, Karolinska University Hospital, Stockholm, Sweden

[4]Department of Emergency Medicine, Karolinska University Hospital, Stockholm, Sweden
